# The Human Serum Metabolome

**DOI:** 10.1371/journal.pone.0016957

**Published:** 2011-02-16

**Authors:** Nikolaos Psychogios, David D. Hau, Jun Peng, An Chi Guo, Rupasri Mandal, Souhaila Bouatra, Igor Sinelnikov, Ramanarayan Krishnamurthy, Roman Eisner, Bijaya Gautam, Nelson Young, Jianguo Xia, Craig Knox, Edison Dong, Paul Huang, Zsuzsanna Hollander, Theresa L. Pedersen, Steven R. Smith, Fiona Bamforth, Russ Greiner, Bruce McManus, John W. Newman, Theodore Goodfriend, David S. Wishart

**Affiliations:** 1 Department of Computing Science, University of Alberta, Edmonton, Canada; 2 Department of Chemistry, University of Alberta, Edmonton, Canada; 3 Department of Clinical Laboratory Medicine, University of Alberta, Edmonton, Canada; 4 Department of Biological Sciences, University of Alberta, Edmonton, Canada; 5 National Institute for Nanotechnology, Edmonton, Canada; 6 James Hogg iCAPTURE Centre for Cardiovascular and Pulmonary Research and the NCE CECR Centre of Excellence for Prevention of Organ Failure (PROOF Centre), Vancouver, Canada; 7 United States Department of Agriculture, Agricultural Research Service (ARS), Western Human Nutrition Research Center, Davis, California, United States of America; 8 Pennington Biomedical Research Center, Baton Rouge, Louisiana, United States of America; 9 Veterans Administration Hospital and University of Wisconsin School of Medicine and Public Health, Madison, Wisconsin, United States of America; Aston University, United Kingdom

## Abstract

Continuing improvements in analytical technology along with an increased interest in performing comprehensive, quantitative metabolic profiling, is leading to increased interest pressures within the metabolomics community to develop centralized metabolite reference resources for certain clinically important biofluids, such as cerebrospinal fluid, urine and blood. As part of an ongoing effort to systematically characterize the human metabolome through the Human Metabolome Project, we have undertaken the task of characterizing the human serum metabolome. In doing so, we have combined targeted and non-targeted NMR, GC-MS and LC-MS methods with computer-aided literature mining to identify and quantify a comprehensive, if not absolutely complete, set of metabolites commonly detected and quantified (with today's technology) in the human serum metabolome. Our use of multiple metabolomics platforms and technologies allowed us to substantially enhance the level of metabolome coverage while critically assessing the relative strengths and weaknesses of these platforms or technologies. Tables containing the complete set of 4229 confirmed and highly probable human serum compounds, their concentrations, related literature references and links to their known disease associations are freely available at http://www.serummetabolome.ca.

## Introduction

Metabolomics is a branch of “omics” research primarily concerned with the high-throughput identification and quantification of small molecule (<1500 Da) metabolites in the metabolome [1], [2]. While in other “omics” fields, including genomics, transcriptomics and proteomics thousands of targets are routinely identified and quantified at a time, the same cannot be said of most metabolomics efforts. Indeed, the majority of published metabolomic studies identify and/or quantify fewer than two dozen metabolites at a time [3]. In other words, metabolomics currently lacks the quantitative horsepower that characterizes the other “omics” sciences. This limitation has mostly arisen because metabolomics has, until recently, lacked the electronic database equivalent of GenBank or UniProt [2] for compound identification. With the release of the Human Metabolome Database (HMDB) [4], [5] and other related compound or spectral resources such as KEGG [6], LipidMaps [7], PubChem [8], ChEBI [9], MMCD [10], Metlin [11] and MassBank [12], we believe the field has taken an important step towards making metabolomics studies much more quantitative and far more expansive in terms of metabolite coverage. In an effort to further enhance the use of quantitative metabolomics, we (and others) have started to systematically determine the detectable metabolic composition of clinically important biofluids and tissue types [13], [14], [15]. Following our comprehensive characterization of the cerebrospinal fluid metabolome [15] we continue herein with a comprehensive characterization of the human serum metabolome.

Blood is composed of two parts: a cellular component consisting of red and white blood cells and platelets, and a liquid carrier, called plasma. Plasma is the straw-colored liquid in which blood cells are suspended, which accounts for approximately 50–55% of blood volume, with blood cells (erythrocytes, leukocytes and platelets) accounting for the remaining portion [16]. Plasma is obtained from a blood sample, if anti-coagulants are introduced, by simply centrifuging the sample and removing or decanting the most buoyant (non-cellular) portion. If no anticoagulant is added and the blood is allowed to clot, the supernatant fluid is called the serum, which is less viscous than plasma and lacks fibrinogen, prothrombin and other clotting proteins [17]. Both plasma and serum are aqueous solutions (about 95% water) containing a variety of substances including proteins and peptides (such as albumins, globulins, lipoproteins, enzymes and hormones), nutrients (such as carbohydrates, lipids and amino acids), electrolytes, organic wastes and variety of other small organic molecules suspended or dissolved in them. In terms of small molecules, the compositions of plasma and serum appear to be very similar (based on current analytical techniques). The primary difference appears to lie in the compounds involved in the clotting process; although modest discrepancies in the relative distribution of some compounds between these pools have also been reported [18] The clotting of blood maximally stimulates blood cell eicosanoid biosynthesis, and thus serum levels of these metabolites do not reflect physiological concentrations [19]. Therefore, due to their clinical importance, measures of plasma eicosanoids have been included in this report. However, to improve readability of the manuscript, the term “serum” is used when referring to the liquid portion of blood, except where explicit measures in plasma are discussed.

Blood serum is a primary carrier of small molecules in the body. Not only does this biofluid play a critical role in transporting dissolved gases, nutrients, hormones and metabolic wastes, but it also plays a key role in the regulation of the pH and ion composition of interstitial fluids, the restriction of fluid losses at injury sites, the defense against toxins and pathogens and the stabilization of body temperature [20]. Because blood bathes every tissue and every organ in the body, it essentially serves as a liquid highway for all the molecules that are being secreted, excreted or discarded by different tissues in response to different physiological needs or stresses. Of crucial clinical importance is the fact that tissue lesions, organ dysfunctions and pathological states can alter both the chemical and protein composition of blood plasma/serum. As a result, most of today's clinical tests are based on the analysis of blood plasma or blood serum [21], [22].

Being an important and easily accessible biological fluid, blood has been the subject of detailed chemical analysis for more than 70 years [21], [23]. Extensive tables of normal reference ranges have been published for many blood gases, ions and about 100 metabolites [22], [24], [25], [26], [27]. In addition to these referential clinical chemistry studies, several groups have applied various “global” metabolomic or metabolite profiling methods, such as high resolution nuclear magnetic resonance (NMR) spectroscopy [28], [29], high performance liquid chromatography (HPLC) [30], amino acid analysis [31], [32] liquid chromatography – mass spectrometry (LC-MS) [33], [34], high performance liquid chromatography – mass spectrometry/mass spectrometry (HPLC-MS/MS) [35], gas chromatography – mass spectrometry (GC-MS) [36], [37], high resolution capillary GC-MS [38], GCxGC-MS [39], ultrahigh performance liquid chromatography – mass spectrometry (UPLC-MS) [40] and high resolution reversed-phase LC (RPLC) with high resolution quadrupole time-of-flight mass spectrometry (QqTOF) [41] to characterize the serum/plasma metabolome with varying degrees of success. Perhaps the most complete global characterization of the blood metabolome to date was described by Lawton and colleagues [13]. Using a combination of GC-MS and LC-MS, this group reported the identification of more than 300 metabolites or metabolic features (of which 79 were explicitly identified) in the human plasma metabolome. A similar GC-TOF-MS study identified nearly 80 low molecular weight metabolites in blood plasma [42], whereas a recent high resolution capillary GC-MS study has provided a very extensive list of lipid fatty acids in blood [38]. In addition to these global metabolomic studies, hundreds of other “targeted” metabolite studies have been conducted on blood plasma and serum that have led to the identification and quantification of hundreds of other serum metabolites. Unfortunately, this information is not located in any central repository. Instead it is highly dispersed across numerous journals and periodicals [4].

To facilitate future research into blood chemistry and blood metabolomics, it is crucial to establish a comprehensive, electronically accessible database of the detectable metabolites in human blood, plasma and/or serum. This document presents just such a database, describing the metabolites that can be detected in human serum (along with signaling molecules in blood plasma), along with their respective concentrations and disease associations. This resource was assembled using a combination of both experimental and literature-based research. Experimentally, we used high-resolution NMR spectroscopy, GC-MS, TLC/GC-MS, LC-MS, UPLC-MS/MS, and direct flow injection (DFI) MS/MS methods to identify, quantify and validate more than 4000 plasma and serum metabolites. To complement these “global” metabolic profiling efforts, our team also surveyed and extracted metabolite and disease-association data from more than 2000 books and journal articles that had been identified through computer-aided literature and in-house developed text-mining software. This “bibliomic” effort yielded data for another 665 metabolites. The resulting Serum Metabolome Database (SMDB) (http://www.serummetabolome.ca) is a comprehensive, web-accessible resource containing these 4229 confirmed and probable serum/plasma compounds, their corresponding concentrations and links to disease associations that were revealed or identified from these combined experimental and literature mining efforts.

In undertaking this study we chose to emphasize breadth over depth. In other words, rather than producing detailed, gender, ethnic or age-specific ranges for hundreds or thousands of patients for a few compounds, we instead produced a broad survey for hundreds or thousands of compounds from a relatively modest number of individuals. While some of the resulting (literature or experimentally derived) concentration values for many of these compounds might not be appropriate for routine clinical studies, they do provide a far more complete and quantitative picture of the plasma/serum metabolome than has previously been achieved. They also provide “ballpark” concentration values for many metabolites that have never been measured or whose concentration values are not widely known. Overall, the intent of this study was to help both the metabolomics and blood research communities address four key questions: 1) What compounds can be or have ever been identified in blood? 2) What are the approximate concentration ranges for these metabolites? 3) What portion of the serum metabolome can be routinely identified and/or quantified using untargeted or “global” metabolomics methods? and 4) What analytical methods (NMR, GC-MS, LC-MS, DFI-MS/MS, etc.) are best suited for comprehensively characterizing the serum metabolome? We believe that answers to these questions provide a more suitable baseline for both future and ongoing blood metabolomic studies (e.g. the HUSERMET study [43] (http://www.husermet.org/). Indeed, such a baseline would allow more prudent selection of appropriate metabolomics platforms and eventually lead to a more complete accounting of age, gender, diet and ethnicity variations.

## Results and Discussion

### The Content of the Human Serum Metabolome – The Serum Metabolome Database

A complete listing of the identity and quantity of endogenous metabolites that can be detected in human serum is available in the Serum Metabolome Database (SMDB: http://www.serummetabolome.ca). This freely available, easily queried, web-enabled database provides a list of the metabolite names, level of verification (confirmed or probable), normal and disease-associated concentration ranges, diseases and references for all (to the best of our knowledge) human serum metabolites that have ever been detected and quantified in the literature. It also contains the concentration data compiled from the experimental studies described here. Each serum metabolite entry in this database is linked to a MetaboCard button that, when clicked, brings up detailed information about that particular entry. This detailed information includes nomenclature, chemical, clinical and molecular/biochemical data. Each MetaboCard entry contains more than 110 data fields many of which are hyperlinked to other databases (KEGG, PubChem, MetaCyc, ChEBI, PDB, Swiss-Prot, and GenBank) as well as to GeneCard IDs, GeneAtlas IDs and HGNC IDs for each of the corresponding enzymes or proteins known to act on that metabolite. Additionally, SMDB through its MetaboCard/HMDB links includes nearly 300 hand-drawn, zoomable and fully hyperlinked human metabolic pathway maps. These maps are intended to help users visualize the chemical structures on metabolic maps and to get detailed information about metabolic processes. These SMDB pathway maps are quite specific to human metabolism and explicitly show the subcellular compartments where specific reactions are known to take place.

SMDB's simple text query (TextQuery) supports general text queries including names, synonyms, conditions and disorders. Clicking on the Browse button (on the SMDB navigation panel) generates a tabular view that allows users to casually scroll through the database or re-sort its contents by compound name or by concentration. Users can choose either the “Metabolite View” or “Associated Condition View” to facilitate their browsing or searching. Clicking on a given MetaboCard button brings up the full data content (from the HMDB) for the corresponding metabolite. The ChemQuery button allows users to draw or write (using a SMILES string) a chemical compound to search the SMDB for chemicals similar or identical to the query compound. ChemQuery also supports chemical formula and molecular weight searches. The TextQuery button supports a more sophisticated text search (partial word matches, misspellings, etc.) of the text portion of SMDB. The SeqSearch button allows users to conduct BLAST sequence searches of the 6252 protein sequences contained in SMDB. Both single and multiple sequence BLAST queries are supported. The DataExtractor button opens an easy-to-use relational query search tool that allows users to select or search over various combinations of subfields. The DataExtractor is the most sophisticated search tool in SMDB. SMDB's MS Search allows users to submit mass spectral files (MoverZ format) that will be searched against the Human Metabolome database (HMDB)'s library of MS/MS spectra. This potentially allows facile identification of serum metabolites from mixtures via MS/MS spectroscopy. SMDB's NMR Search allows users to submit peak lists from ^1^H or ^13^C NMR spectra (both pure and mixtures) and to have these peak lists compared to the NMR libraries contained in the HMDB. This allows the identification of metabolites from mixtures via NMR spectroscopy. The Download button provides links to collected sequence, image and text files associated with the SMDB. The Explain button lists source data used to assemble the SMDB.

Currently the SMDB contains information on 4229 *detectable* metabolites (both confirmed and probable) and 9225 concentration ranges or values associated with different conditions and disorders. This is not a number that will remain unchanged. Rather it reflects the total number of metabolites – most of which are endogenous - that have ever been detected and quantified by others and ourselves. Certainly as technology improves, we anticipate this number will increase as other, lower abundance, metabolites, are detected and added to future versions of the SMDB. Likewise, if the list was expanded to include intermittent, exogenous compounds such as all possible drugs or drug metabolites or rare food additives and food-derived phytochemicals, the database could be substantially larger.

Inspection of the on-line tables in SMDB generally shows that human serum contains a substantial number of hydrophobic or lipid-like molecules. This is further emphasized in Table 2, which provides a listing of the metabolite categories in human serum and the number of representative compounds that can be found in this biofluid. Overall, the composition of human serum is dominated by diglycerides, triglycerides, phospholipids, fatty acids, steroids and steroid derivatives. This simply reinforces the fact that serum (i.e. blood) is a key carrier of lipoproteins, fats and hydrophobic nutrients. Other small molecule nutrients found in high abundance in serum include amino acids (10 µM–1155 µM), glucose, glycerol, lactate, oxygen, carbon dioxide (in the form of bicarbonate ions) and several waste or catabolic byproducts such as urea and creatinine. A more detailed description of our findings is given in the following 6 sections covering: 1) Literature Review/Text Mining; 2) NMR; 3) GC-MS; 4) LC-ESI-MS/MS Targeted Lipid Profiling; 5) Lipidomics via TLC/GC-FID; and 6) DFI MS/MS.

### Metabolite Concentration in Serum – Literature Survey

In addition to the experimentally derived values obtained for this study, the serum metabolome database (SMDB) also presents literature-derived concentrations of the metabolites with references to either PubMed IDs or to clinical texts. In many cases, multiple concentration values are given for “normal” conditions. This is done to provide users/readers with a better estimate of the potential concentration variations that different technologies or laboratories may measure. As a general rule, there is good agreement between most laboratories and methods. However, the literature results presented in the SMDB do not reflect the true state of the raw literature. A number of literature-derived concentration values were eliminated through the curation process after being deemed mistaken, disproven (by subsequent published studies), mis-typed or physiologically impossible. Much of the curation process involved having multiple curators carefully reading and re-reading the primary literature to check for unit type, unit conversion and typographical inconsistencies.

Other than the inorganic ions and gases such as sodium (144 mM), chlorine (110 mM), bicarbonate/carbon dioxide (25 mM), iron (9 mM), oxygen (6 mM), potassium (4.5 mM), calcium (2.5 mM), phosphorus/phosphate and sulfur/sulfide (∼1 mM) and magnesium (800 µM), the 12 most abundant organic metabolites found in serum are D-glucose (5 mM), total cholesterol (5 mM), melanin (5 mM), urea (4 mM), ATP (3 mM), glyceraldehyde (1.5 mM), cholesterol esters (0.4–1.5 mM), L-lactic acid and fructosamine (∼1 mM), L-glutamine (500 µM), L-alanine (500 µM), methanol (460 µM), glycine, L-lysine, uric acid (350 µM), and (R)-3-hydroxybutyric acid (350 µM). The least abundant (detectable) metabolites in serum include several diacylglycerols (DGs), (>1 pM), LPS-o-antigen (2pM), vitamin K1 2,3-epoxide (2 pM), 13,14-dihydro prostaglandin E1 (PGE1) (3 pM), substance P and prostaglandin E1 (4 pM), various glycerophospholipids (4–100 pM), vasopressin (5 pM), 11-trans-Leukotriene C4 (10 pM), nitric oxide (12 pM), LPS core (14 pM), thyroxine (15 pM), 3,5-diiodothyronine (16 pM), epietiocholanolone, thromboxane B3 and cotinine N-oxide (17 pM), thyroxine sulfate and 11b-hydroxyprogesterone (∼20 pM). This shows that the current lower limit of detection for metabolites in serum is in the low picomolar range and that the concentration range of analytes in serum spans nearly 11 orders of magnitude. As might be expected, many of the least abundant compounds are hormones or signaling molecules while the most abundant molecules serve as buffering agents or stabilizing salts.

One point that is particularly interesting is the fact that the concentration of the average metabolite in normal serum varies by about +/−50%, with some metabolites varying by as much as +/−100% (such as D-glucose, L-lactic acid, L-glutamine, glycine). Therefore, drawing conclusions about potential disease biomarkers without properly taking into account this variation would be ill-advised. We believe that these relatively large ranges of metabolite concentrations are due to a number of factors, including age, gender, genetic background, diurnal variation, health status, activity level, and diet. Indeed, some SMDB entries explicitly show such variations based on the populations (age, gender) from which these metabolite concentrations were derived. Clearly more study on the contributions to the observed variations in serum is warranted, although with thousands of metabolites to measure for dozens of conditions, these studies will obviously require significant technical and human resources.

### Experimental Quantification and Identification – NMR

**Figure 1:**
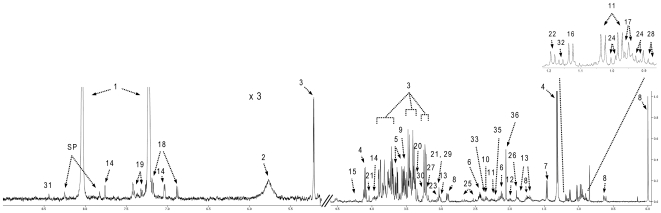
Typical 500 MHz ^1^H-NMR spectrum of healthy human serum. Numbers indicate the following metabolites: 1, imidazole; 2, urea; 3, D-glucose; 4, L-lactic acid; 5, glycerol; 6, L-glutamine; 7, L-alanine; 8, DSS; 9, glycine; 10, L-glutamic acid; 11, L-valine; 12, L-proline; 13, L-lysine; 14, L-histidine; 15, L-threonine; 16, propylene glycol; 17, L-leucine; 18, L-tyrosine; 19, L-phenylalanine; 20, methanol; 21,creatinine; 22, 3-hydroxybutyric acid; 23, ornithine; 24, L-isoleucine; 25, citric acid; 26, acetic acid; 27, carnitine; 28, 2-hydroxybutyric acid; 29, creatine; 30, betaine; 31, formic acid; 32, isopropyl alcohol; 33, pyruvic acid; 34, choline; 35, acetone; 36, glycerol.

The NMR spectrum of ultrafiltered serum is remarkably simple and surprisingly uncomplicated (Figure 1) This made the identification and quantification of serum metabolites relatively easy. Typically 98% of all visible peaks were assigned to a compound and more than 95% of the spectral area could be routinely fitted using the Chenomx spectral analysis software. As seen in Figure 1, most of the visible peaks can be annotated with a compound name. From the 21 healthy control serum samples and the 54 serum samples from the patient cohort, 20 and 53 were analyzed, respectively. A total of 44 unique compounds were identified with an average of 33±2 compounds being identified per sample. Twenty-five compounds were identified in every sample, with the most abundant compounds being urea (6 mM), D-glucose (5 mM), L-lactic acid, (1.4 mM), L-glutamine (0.51 mM) and glycerol (0.43 mM). The least abundant compounds were carnitine (46 µM), acetic acid (42 µM), creatine (37 µM), L-cysteine (34 µM), propylene glycol (22 µM) and L-aspartic acid (21 µM). The lowest concentration that could be reliably detected using NMR was 12.3 µM (for malonic acid) and 14.5 µM (for choline). The complete list of average compound concentrations and the frequency of their occurrence is shown in Table 3. Significant efforts were made to identify the “rarer” or less frequently occurring compounds in a larger fraction of serum samples. To this end, we collected NMR spectra for longer periods of time and/or at higher field strengths. While this did improve quantification accuracy, it did not lead to an increase in the number of compounds detected. Inspection of Table 3 also reveals the generally good agreement between the NMR-measured concentrations and those reported in the literature (often obtained by other analytical means).

However, not all of the NMR-derived serum concentrations agree with the literature derived values. Forty-three out of the 44 compounds identified in the healthy control group, had concentration values previously reported in the literature. We found that 35 compounds exhibited good agreement with literature values –i.e., meaning the average NMR values fell within one standard deviation of the literature value. In addition, seven compounds had concentrations somewhat higher than previously reported values (L-asparagine, glycerol, glycine, L-histidine, hypoxanthine, methanol and propylene glycol), while two compounds had concentrations lower than previously reported (L-cystine and formic acid). Compounds exhibiting the greatest discrepancy between NMR measured values and literature derived values include: glycerol, hypoxanthine and propylene glycol. Propylene glycol is likely an exogenous “contaminant” as it is widely used as a solvent in many pharmaceuticals and as a moisturizer in cosmetics, lotions, hand sanitizers, foods and toothpastes. Nevertheless, its ubiquity in so many consumer products has made it a routinely observed component of human serum. Some of the concentration discrepancies for the other compounds may be explained by their inherent volatility or chemical instability (hypoxanthine, methanol, formic acid). Other discrepancies may be due to sample collection/preservation effects (the ultrafiltration devices we used contain trace amounts of glycerol) or possibly sample size effects (2 patients versus 35 patients). A third source of variation may be technical problems with the separation or extraction methods being used to obtain “clean” serum by different laboratories. Blood is an inherently complex, multi-component mixture and organic solvent extractions and ultrafiltration methods have different weaknesses. In particular, while solvent extraction will only isolate soluble components, ultrafiltration will only isolate compounds not tightly associated with macromolecules.

In contrast to the healthy controls, the NMR spectra of the serum isolated from heart transplant patients tended to be slightly more complex and somewhat more variable. A total of 44 compounds were identified from these samples with an average of 32±2 compounds being identified per sample. Twenty-one compounds were identified in every sample. The same level of spectral coverage (98% peak identification, 95% spectral area) was achieved with serum from the heart transplant samples as with the healthy controls. While the rank order of the most abundant and least abundant compounds was slightly different, the same compounds appeared in both the “diseased” and “healthy” lists. The complete list of average compound concentrations for the heart transplant patients along with their frequency of occurrence is shown in Table 3. Inspection of Table 3 again shows the generally good agreement between the NMR-measured concentrations and those reported in the literature, although there are clear and statistically significant differences between the average values for the transplant patients and the normal or literature derived values. Using a Student's t-test we found that 22 compounds had concentrations significantly different between the two groups (using a cut-off P-value of 0.05; with no Bonferroni correction). The most strongly differentiating compounds were D-glucose, creatinine, L-valine, propylene glycol, citric acid, formic acid and L-alanine (data not shown).

Serum from heart transplant patients also provided an opportunity to look at the longitudinal or temporal metabolite variation in individuals. Table S1 summarizes the mean concentration and standard deviation seen over the 12-week sampling period for the 44 metabolites as measured for all 9 transplant patients. Interestingly, the cross-sectional variation appears, in general, to be larger than the longitudinal variation. In other words, time-dependent metabolite changes within a single individual tend to be smaller than the differences seen between different individuals. Furthermore, in a recent analysis of the adult human plasma metabolome, it was found that the concentrations of about 30 metabolites can vary more than 50% between healthy individuals due to age, gender and body mass index [13]. These temporal variations may be somewhat exaggerated over what might be seen in the general population given the surgical trauma and medication that these heart transplant patients have experienced over the sampling period. Nevertheless, the objective of this study was to gain a better idea of the variability of serum metabolite concentrations that can be found in living adults (without an obvious metabolic disorder).

Combining the complete set of results from the healthy control subjects with the results from the heart transplant patients, we were able to identify and quantify a total of 49 different compounds in serum using NMR spectroscopy (Table 3). We would argue that this list of 49 metabolites along with the concentrations listed in this table defines the “normal NMR-detectable serum metabolome”. Furthermore, we believe that now that this set of 49 metabolites is known, it should allow the NMR characterization of unprocessed serum to become routine, if not highly automated.

### GC-MS Identification and Quantification

**Figure 2:**
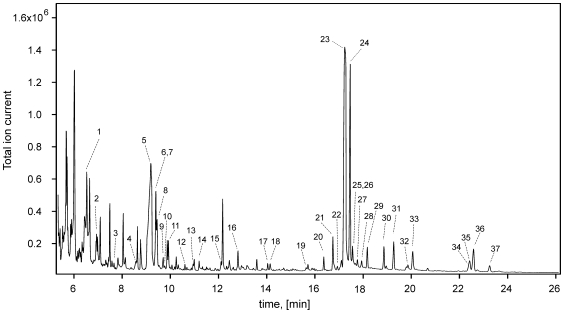
Typical total ion chromatogram of serum from a healthy subject. Numbers indicate the following metabolites: 1, L-lactic acid; 2, L-alanine; 3, oxalic acid; 4, L-valine; 5, urea; 6, L- L- L-leucine; 7, glycerol; 8, phosphoric acid; 9, L-isoleucine; 10, L-proline; 11, glycine; 12, L- L- L-serine; 13, L-threonine; 14, L-methionine/L-aspartic acid; 15, aminomalonic acid; 16, pyroglutamic acid/L-glutamine; 17, L-glutamic acid; 18, L-phenylalanine; 19, L-ornithine; 20, citric acid; 21,d-erythrofuranose; 22, D-fructose; 23, D-glucose; 24, D-galactose; 25, L-histidine; 26, L-lysine; 27, L-tyrosine; 28, gulonic acid/mannonic acid; 29, D-glucopyranose; 30, 6-deoxy mannose; 31, palmitelaidic acid; 32, palmitic acid; 33, myo-inositol; 34, uric acid; 35, L-tryptophan; 36, linoleic acid; 37, oleic acid; 38, stearic acid.

Figure 2 illustrates a typical high resolution GC-MS total ion chromatogram of the polar extracts from a serum sample of a healthy adult subject. As can be seen in this figure, many of the visible peaks can be annotated with a compound name, however ∼40% of these peaks remain unidentified. This relatively low level of coverage is a common problem in global or untargeted GC-MS metabolomics studies. While some of these peaks may be due to derivatization by-products or degraded metabolites, the lack of a comprehensive GC-MS library for human metabolites (the NIST mass spectra library contains only a small portion of metabolically relevant compounds), also limits the attainable coverage from automated library search algorithms. The use of other commercially available reference libraries for GC-MS (i.e. the Fiehn GC-MS library from Agilent) might have provided a slightly more complete coverage of the serum metabolites, and the routine application of more comprehensive libraries will likely expand the list of commonly identified metabolites in the future.

All peaks corresponding to an identified metabolite were verified with pure standards and correlated to literature values. In total we identified 62 polar metabolites and 12 nonpolar metabolites via GC-MS (Table 4). For full identification, the mass spectra of the identified compounds not only had to match the EI-MS spectra in the NIST database (with a match factor of >60% and a probability score >20%), but also the retention index (RI) of the compounds in the University of Alberta RI library, which consists of 312 TMS-derivatized human metabolites. The targeted GC-MS analysis for non-esterified fatty acids in the plasma collected at the Pennington Biomedical Research Center (PBRC) resulted in the identification and quantification of 25 compounds (Table 5) in all samples. Trace levels of other fatty acids were observed, but these were observed intermittently and the signal-to-noise ratio was low enough that these compounds were not deemed of sufficient quality to report. Of the detectable compounds, 9 fatty acids were identified by both targeted and global GC/MS analysis, while 2 were unique for the global analysis (capric acid and arachidic acid) and 16 were unique for the targeted approach. Notably, capric acid (C10:0) was compromised by interference in the PBRC samples, while arachidic acid (C20:0) was observed at <0.05% of the total fatty acid profile in ∼60% of the subjects. Collectively, if both global and targeted GC-MS analyses are taken into account, the number of identified compounds is 90. Of the 74 polar metabolites identified in the global GC-MS approach, only 33 could be clearly quantified. This included 14 additional metabolites that were not detected/quantified by NMR but that could be quantified by GC-MS using external calibration methods (Table 6). Among these compounds, oxalic acid and uric acid were found to have concentrations greater than 1 standard deviation above previously reported values. Comparisons between the NMR and GC-MS measured concentrations (across the 26 compounds that were quantified by both techniques) show generally good agreement (within 20–50% of each other). GC-MS methods typically gave higher concentrations of L-glutamic acid, L-isoleucine, L-methionine and citric acid, and lower concentrations of L-alanine, L-glutamine, glycine, L-serine, glucose and glycerol, compared to the corresponding NMR results (data not shown). Compound concentrations below 1 µM were associated with low signal-to-noise ratio responses, limiting accurate quantification. However these compounds were identified based on previously described methods [69].

For GC-MS, the lowest limit of quantification was 8.3 µM for alpha-hydroxyisobutyric acid. Given that there are slightly over 260 compounds in the serum-metabolome database (SMDB) with normal concentrations >1 µM, one might have expected that the GC-MS detectable compounds would have been much higher than 90. The use of a relatively slow scanning quadrupole instrument may partially explain the limited number of identifiable peaks. This hardware can yield insufficient sampling across co-eluting GC peaks, limiting complex spectral deconvolution. However, comparisons to other reports on serum analysis by GC-MS instruments suggest that this number is not unreasonable. Indeed, a GC-MS (TOF) study on human plasma performed by Jiye et al. yielded a list of 80 metabolites [42]. Our results, using a less-sensitive GC-quadrupole-MS instrument, yielded 90 metabolites (of which 57 were common to both studies). This is primarily because we employed both polar and non-polar extraction techniques to effectively increase the concentration of certain metabolites. No doubt the use of better instruments (i.e. faster scanning quadrupoles or TOF instruments with greater sensitivity), multiple extraction steps or the use of different derivatization steps could have improved compound coverage. Indeed, in a recently published study of the adult serum metabolome, the use of fast-scanning quadrupole GC-MS resulted in the detection (but not the quantification) of about 120 compounds [13]. It is also notable that the authors of this study used a series of four solvent extraction steps compared to the two solvent extraction protocol used in our study. These differences in metabolite numbers may also reflect intrinsic differences in the GC–MS deconvolution software and protocols.

Unlike NMR, where no chemical reactions or extractions are required, GC-MS techniques can lead to the detection of artifactual metabolites. For instance, one of the 76 metabolites reported by Jiye et al. [42] was butylamine. In our study, butylamine was also detected. However it was seen in both human serum and in control (blank) samples. This strongly suggests that butylamine is more an artifact of chemical derivatization, and not a serum metabolite as originally reported. As we previously noted, approximately 40% of the peaks remain unidentified in our GC–MS analyses. These unidentified peaks in the total ion chromatogram were generally of low intensity, making spectral identification difficult. Nevertheless, several standards were run to confirm retention times and mass spectral information, likewise, other GC–MS metabolome libraries were also queried to identify these ‘unknown” peaks, but without success.

**Figure 3:**
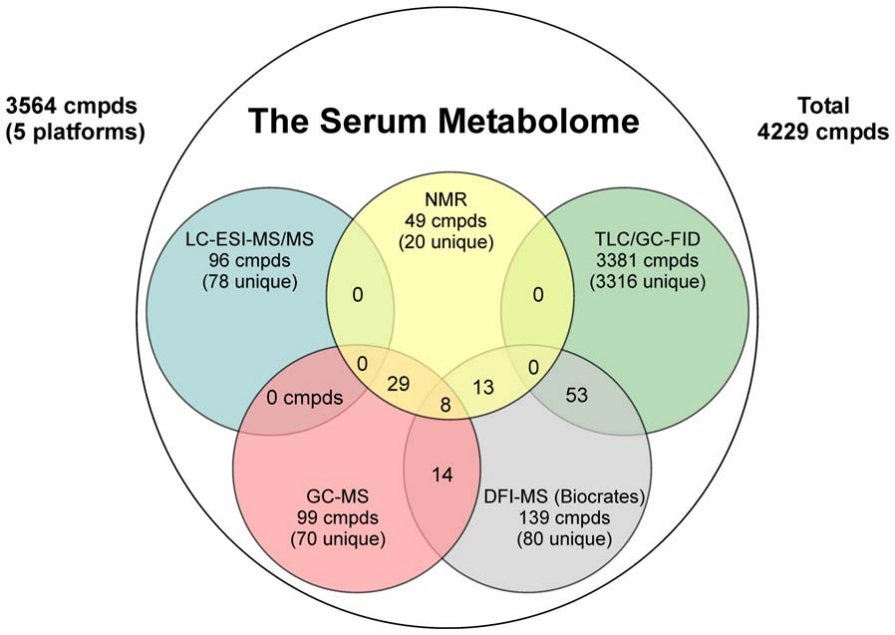
Venn diagram showing the overlap of serum metabolites detected by global NMR, GC–MS, LC/GC-FID, LC-ESI-MS/MS and MS/MS methods compared to the detectable serum metabolome.

It is also of some interest to compare the results of our GC-MS studies with our NMR studies. As seen in Table 3 (NMR results), Table 4–[Table pone-0016957-t005] Table 6 (GC-MS results), and Figure 3, NMR and GC-MS methods identify a common set of 29 compounds. Interestingly, NMR detects 20 compounds that GC-MS methods cannot detect while GC-MS detects 45 compounds that NMR cannot routinely detect, including 3 very high abundance compounds (phosphoric acid/phosphate, uric acid and N-acetyl-glycine). There are many possible reasons for these differences in instrumental detection. A compound might be found by NMR, but not by GC-MS, if it is too volatile/nonvolatile for GC-MS detection, lost in sample preparation or eluted during the solvent delay. A compound might be found by GC-MS, but not by NMR, if its protons are not detectable by NMR (uric acid, phosphate), or if its concentration is below detectable limits (maltose, ribitol). In all cases, the existence of NMR detectable metabolites was explicitly checked in our GC-MS analyses and vice versa. Together, NMR with targeted and global GC-MS identified and quantified 135 mostly polar metabolites. Overall, GC-MS and NMR appear to be complementary techniques for the identification and quantification of small molecules in human serum.

### UPLC-ESI-MS/MS (Targeted Lipid Profiling) Identification and Quantification

While untargeted or global NMR and scanning MS techniques are particularly useful for the identification and quantification of polar metabolites of moderate abundance, they are not well suited for low-abundance metabolites. On the other hand, LC-MS methods are superb at the targeted identification of low-abundance metabolites over a wide polarity range. To exploit and explore these strengths in LC-MS, we chose to study an array of metabolic products of polyunsaturated fatty acids found in the liquid portion of the blood. In particular, we targeted (identified and quantified) a subset of oxylipins (n = 76), acyl-glycines (n = 2), acyl-ethanolamides (n = 12), and mono-acylglycerols (n = 6).

Oxylipins constitute a broad structural class of oxidized lipid molecules, occurring in the low nM to µM concentrations that perform a variety of functions when found in appropriate contexts [52]. Acyl-amides and mono-acyl glycerols are also common components of blood with an equally broad spectrum of actions [70]. These functionalized lipids play important roles in regulating cell proliferation, apoptosis, tissue repair, blood clotting, blood vessel permeability, inflammation, pain perception, pancreatic function, and energy regulation at various levels [71], [72], [73], [74], [75].

The oxylipins are classically formed from polyunsaturated fatty acids through at least three different classes of enzymes: cyclooxygenases (COX-1 and COX-2), lipoxygenases (LOX) and cytochrome P450s, or through the direct interaction between unsaturated lipids and reactive oxygen. The reactive oxygen itself may have either enzymatic (e.g. meyloperoxidase) or non-enzymatic sources [76]. Among the recognized mammalian oxylipins are the arachidonic acid-derived prostaglandins, leukotrienes, lipoxins, hepoxilins, hydroxy, epoxy and dihydroxy metabolites as well as analogs formed from other highly unsaturated lipids (e.g. resolvins, protectins), and an array of oxygenated eighteen carbon lipids.

The acyl-ethanolamides and 2-acyl glycerols have emerged as important endogenous ligands of the cannabinoid receptors, vanilloid receptors, and peroxisome proliferator activated receptors, and their regulated synthesis and degradation impacts satiety, thermogenesis, pain perception, and lipid metabolism [57], [77], [78] In addition, circulating levels of acylethanolamides, but not 2-arachidonyl glycerol, are elevated in cirrhotic liver disease [79] and altered by psychosocial stress [80]. Interestingly, cross talk between the acyl-ethanolamine and oxylipin pathways have also been reported [81]. While less well studied, the acyl-glycines represent a growing class of “orphan” endogenous lipids which are candidate ligands for a variety of orphan G-protein coupled receptors [82].

Within each of these classes of lipid mediators there exists an array of isomeric products from of a relatively few fatty acid species making their separation critical for accurate quantification. Collision induced dissociation (CID) often yields extensive compound fragmentation with structurally unique information that aids identification but limits sensitivity. However, many of these metabolites are present in nM concentrations, thus detection and quantification tasks are even more challenging. Over the past two decades GC-MS, LC-MS and LC-MS/MS methods have all been used to detect, identify and quantify oxylipins and other polar metabolites, however the latter approach is associated with simpler sample workup strategies and can simultaneously assess a broader range of targets [83]. While knowledge of normal circulating ranges of some of these mediators may be valuable, the challenging nature of their detection and quantification has resulted in limited reporting of their circulating concentrations in the literature. Given the paucity of such data, we decided to undertake this targeted study. Not only would the results provide new and useful information for the serum/blood metabolomics community, they would also give a useful assessment of the comparative strength of targeted LC-MS/MS relative to untargeted methods in quantitative metabolomics.

Seventy plasma samples collected at the Pennington Research Center and a triplicate sample (1 sample partitioned into 3 samples) collected by the Human Metabolome Project were analyzed for subsets of lipid mediators at the USDA-ARS-Western Human Nutrition Research Center. Surrogate recoveries were acceptable and are summarized in Table S2. Replicate analysis of a laboratory reference serum (n = 7) analyzed in conjunction with the Pennington Research Center samples showed excellent precision, 72% of the oxylipins and 67% of the acyl-glycerol/amides showed relative standard deviations of <30% for analytes with a signal-to-noise ratio >2.

The negative mode UPLC-(−) ESI/MS/MS analysis resulted in the identification and quantification of 76 oxylipins, including 55 20-carbon polyunsaturated fatty acid-derived oxylipins (Table 7 and Table 8) and 21 18-carbon polyunsaturated fatty-acid-derived oxylipins (Table 9). The positive mode analysis UPLC-(+)ESI/MS/MS analysis resulted in the identification and quantification of 20 acyl-ethanolamides, acyl-glycerols, and acyl-glycines (Table 10). Collectively, the 2 datasets provide information on 76 oxylipins, 12 acyl-ethanolamide, 6 mono acyl-glycerols, and 2 N-acyl glycines.

While serum and plasma are similar with regards to the concentration and composition of many small molecules, it is noteworthy that the physiological concentrations of thromboxanes in serum and plasma differ greatly. Serum is produced by allowing whole blood to clot and coagulate, while plasma is the unclotted liquid fraction of blood. The act of clotting is triggered by platelet degranulation, which releases thromboxane A2 (TXA2) into the blood, initiating the clotting response. TXA2 is unstable in aqueous solution, and is hydrolyzed rapidly into the stable and inactive thromboxane B2 (TXB2), which reflects TXA2 production and platelet activation. Therefore, normal plasma TXB2 levels are very low and range from 0.2 to 2 ng/mL [84]. However, when blood is allowed to naturally clot, then thromboxane production increases considerably and its physiological concentration in the resulting serum has been reported to range from 2 to 178 ng/mL [85].

On the other hand, it is important to mention that non-esterified fatty acids are well-described circulating components of human plasma and are influenced by the fed/fasted state, as well as the metabolic health of the individual. In this regard, it is noteworthy that the analysis of the plasma sample from the Human Metabolome Project showed very high long chain n3-oxylipins, suggesting that this sample was from a person that consumes high amounts of fish or ingests fish oil supplements. This is a nice contrast with respect to the Pennington cohort and indicates the important role that dietary habits play in the oxylipin composition of blood.

### TLC/GC-FID Lipid Analysis

The identification and quantification of a wide array of lipid class isomers within a single analytical sample (i.e. lipidomics) is a rapidly developing sub-field of metabolomics [86], [87]. There are essentially two approaches for identifying and/or quantifying lipids. One approach, known as “shotgun” lipdiomics [88], [89], uses LC-MS techniques to separate lipid classes and mass fragment libraries to identify lipid types. Shotgun lipidomics is a powerful, non-targeted metabolomic technique as it allows lipids to be rapidly and “approximately” identified and/or quantified (if isotopic standards are available). Approximate identification means that a lipid might be identified as PC(38:4), meaning that it is a phosphatidylcholine with two acyl chains that have a total of 4 unsaturated bonds. However, the length of the individual acyl chains, the sn1/sn2 position of the acyl chains and the position of the unsaturated bonds is not generally known nor easily knowable. Indeed, the PC(38:4) designation still means that the lipid could be one of nearly a dozen possible PC structures.

An alternative and much more time-consuming approach to lipidomics involves separating lipid classes individually, quantifying the lipid classes, hydrolyzing the lipids into their constituent acyl chains and then identifying the fatty acids using GC-MS. This method, which is used by Lipomics Technologies Inc. (now Tethys Biosciences, Inc.) as well as other, more “traditional” lipid analysis labs, is more quantitative and allows the length and identity of individual acyl chains to be identified. However, it is not readily amenable to identifying or quantifying the original or intact lipid. We chose to use this latter approach, partly because of its quantitative nature and the fact that combinatorial lipid reconstruction (CLR) could be used to computationally regenerate precise lipid structures and to approximate concentration ranges.

The data generated by Lipomics Technologies Inc. for the three adult serum samples yielded an average number of 26 (ranging from 23 to 32) unique acyl chains that could be identified and quantified, comprised of saturated, monounsaturated, polyunsaturated (ω-3, ω-6, ω-9, plasmalogen) fatty acids. These acyl chains were further distributed among 7 distinct lipid classes: 1) cholesterol esters; 2) diacylglycerols; 3) lysophosphatidylcholines; 4) phosphatidylcholines; 5) phosphatidyl-ethanolamines; 6) free fatty acids and 7) triacylglycerols. Lipids with more than one fatty acid chain (phosopholipids, diacyl and triacylglycerols), had their identities and concentrations determined using combinatorial lipid reconstruction (CLR, see File S1). CLR uses the fractional abundance of each fatty acid chain and the total concentration of a given lipid class to estimate the most probable and upper-limit concentrations of specific lipids. CLR simplifies to solving a linear algebra problem with pre-defined constraints thereby allowing one to estimate most probable and upper limit concentrations. The most probable concentration corresponds to the concentration a given lipid is most likely to have, based on the fractional abundance of all fatty acid components measured for its parent lipid class. The upper limit concentration corresponds to the highest possible concentration for a given lipid assuming no other fatty acid combinations contribute to its total concentration (the code for lipid quantification is briefly described in File S1).

Using both direct measurements (for CE-esters, free fatty acids and lysolipids) and CLR (for phospholipids, diacyl and triacylglycerols), we identified and quantified (or semi-quantified) 3,381 lipids. This total included: 25 “confirmed” cholesterol esters, 27 “confirmed” free fatty acids, 30 “confirmed” lysophosphatidylcholines (Table 11), 847 “probable” diacylglycerols, 1092 “probable” phosphatidylcholines, 1071 “probable” phosphatidylethanolamines, and 289 “probable” triacylglycerols (the most abundant ones). The lower limit of quantification of LC/GC-FID based on the TrueMass® platform and CLR estimates was 9.8 nM for the diacylglycerol known as (Z,Z)-13,16-docosadienoic acid.

Comparison of the TLC/GC-FID lipid results with literature data was difficult as relatively few papers report lipid concentration data for serum and/or plasma. We did find data for a number of total fatty acids, which showed good agreement with the data generated by Lipomics Technologies Inc, as seen by comparison with a cross-sectional study of Kuriki et al [90] in Table S3. Likewise, total cholesteryl ester concentrations, as opposed to individual cholesterol esters, also showed generally good agreement with cholesterol measurements reported in the literature (Table S4). It was also challenging to compare the TLC/GC-FID lipid results with the GC-MS results as the two methods only identified and quantified 8 metabolites in common (arachidonic acid, eicosanoic acid, linoleic acid, oleic acid, palmitelaidic acid, palmitic acid, stearic acid and tetradecanoic acid). Nevertheless, the concentration data showed generally good agreement, with only palmitic acid and oleic acid being substantially different (TLC/GC-FID concentrations were 50% lower for palmitic acid and 60% lower for oleic acid). On the other hand, comparison of the non-esterified or free fatty acids quantitative results between the TLC/GC-FID and the GC-(+)EI MS platforms shows that the GC-(+)EI MS concentrations of palmitic acid, vaccinic acid, oleic acid, linoleic acid, dihomo-γ-linolenic acid and docosapenta-(4,7,10,13,16)-enoic acid are generally higher than those measured by TLC/GC-FID (Table 6 and Table 11). However, as these were measured in different subjects, these differences are likely due to variation in subjects as opposed to methodological inconsistencies. A more detailed comparison of the CLR-derived lipid concentrations to those obtained from other MS/MS methods is given below.

### DFI MS/MS

The Direct Flow Infusion (DFI) MS/MS targeted analysis using the Biocrates Absolute*IDQ* kit provided quantitative results for 139 metabolites (24 acylcarnitines. 14 amino acids, hexose (Table 12), 73 phospatidylcholines (Table S5), 15 sphingomyelins and 12 lysophosphatidylcholines (Table 13). From the 41 measured acylcarnitines, 24 provided quantitative data, whereas the remaining 17 were below the limit of detection (LOD). This result is in good agreement with previous studies conducted by Biocrates (Application Note 1001-1), which indicated a typical pool of human plasma from healthy people yields an average of 23 acylcarntines below the limit of detection.^1^ That note also reported that the concentrations of 5 lysophosphatidylcholines in pooled normal human plasma are typically below the normal LOD, whereas in the present study only 3 lysophosphatidylcholines were below the LOD. In our hands, the lower limit of quantification by DFI MS/MS based on the Absolute*IDQ* kit was 12 nM for hexadecadienylcarnitine.

The Biocrates DFI MS/MS approach generates lipid data that is more akin to that measured via shotgun metabolomics (see above). That is, the lipids are identified by their total acyl/alkyl chain content (i.e. PC(38:4)) as opposed to their precise chemical structure. As a result it was difficult to compare lipid concentration measurements between the Biocrates *IDQ* platform and the Lipomics Technology Inc. platform. Nevertheless, by grouping the diacyl PCs generated by CLR to match the PC designations generated by Biocrates we were able to create a modest correspondence. We found that the concentration data measured by the Biocrates kit, by Quehenberger et al. [91] and by the Lipomics platforms matched reasonably well, with the exception of three cases: 1). PC(28:1), PC(30:2) and PC(38:0) for which CLR estimated considerably lower most-probable concentrations than Biocrates; 2). PC(32:0) and PC(36:0) for which CLR estimated considerably higher; than Biocrates and Quehenberger et al.; and 3) the quantified concentration of PC(40:2) by Quehenberger et al. [91] which is significantly higher than both Biocrates and CLR (Table S6). These discrepancies may be due to the fact that different subjects were analyzed by DFI MS/MS, TLC/GC-FID and LC-MS/MS. In addition, since CLR takes into account all possible sn1/sn2 structural combinations, some of these combinations are unavoidably less likely to exist in nature and so these PCs may be over-represented and therefore generate higher concentrations. On the other hand, the limits of quantification of the three platforms are not identical. This means they quantify different individual fatty acids per lipid class and so they return different numbers of positional combinations and different PC concentrations.

We used similar re-groupings to compare the lysoPC values to each other and found that both the Lipomics and Biocrates platforms provide quite comparable quantification data (Table 14). Overall, while the compound overlap is relatively small, it appears that both platforms provide reliable and closely agreeing measurements of the lipid content in serum.

### Method Comparison

We used five different metabolic profiling methods to experimentally characterize as much of the known serum metabolome as possible: 1) NMR; 2) GC-MS; 3) lipid mediators by LC-ESI-MS/MS; 4) lipidomics profiling via TLC/GC-FID-MS; and 5) DFI MS/MS. We were able to identify a total of 3564 distinct metabolites including several exogenous compounds such as propylene glycol and acetaminophen. NMR spectroscopy was able to identify and quantify 49 compounds, GC-MS was able to identify 90 and quantify 33 compounds, lipid mediator profiling (targeted ESI-MS/MS) identified and quantified 96 compounds, TLC/GC-FID-MS identified and quantified 3381 compounds while DFI MS/MS identified and quantified 139 compounds. NMR and GC-MS were able to identify a common set of 29 metabolites while NMR, GC-MS and DFI MS/MS were able to identify a common set of 15 metabolites (14 amino acids and hexose/glucose). Likewise DFI MS/MS and lipidomics profiling (TLC/GC-FID-MS) could identify a common set of 53 metabolites. This is summarized in the Venn diagram in Figure 3. These differences in metabolite coverage arise because of many reasons, including separation difficulties, sensitivity differences, instrument detection differences, targeted vs. non-targeted methods, compound stability, compound solubility, compound volatility, etc.

**Figure 4:**
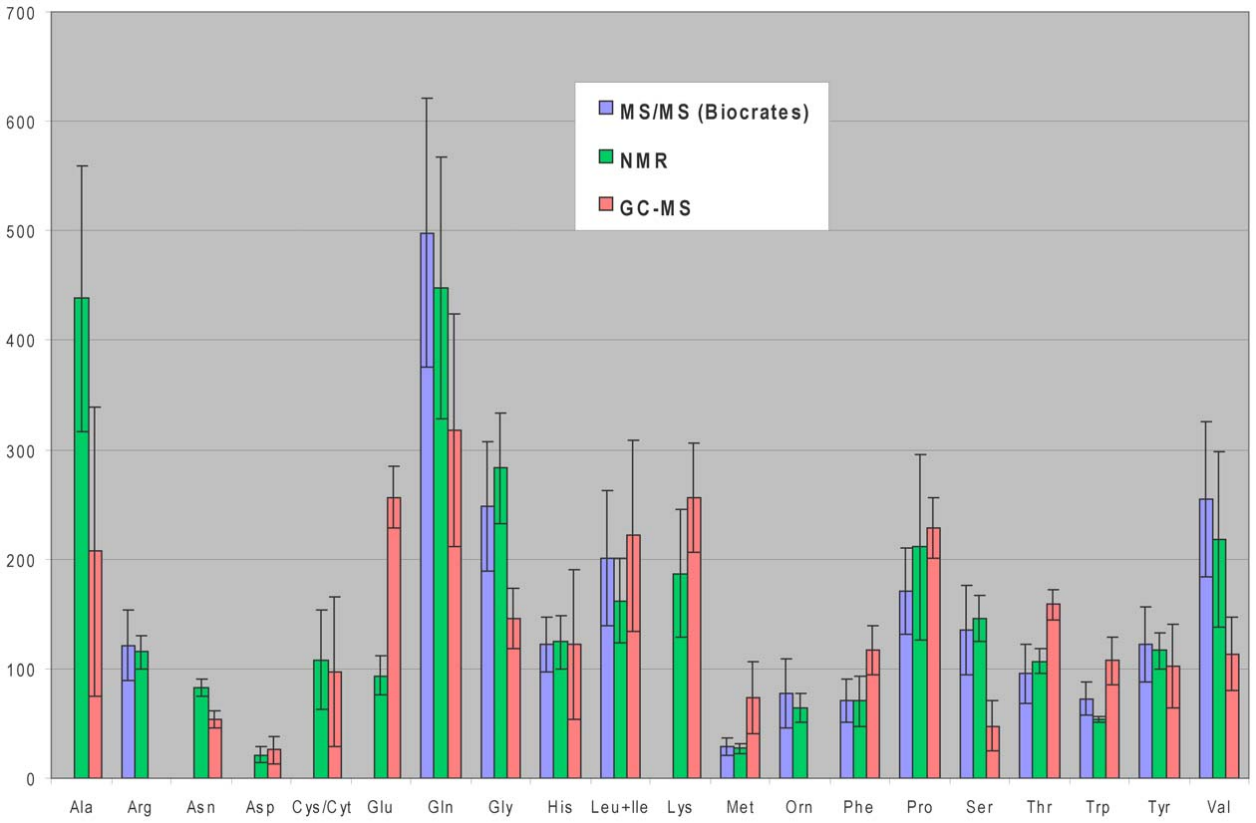
Graphical representation of serum concentrations of amino acids by NMR, GC/MS and MS/MS (Biocrates kit). The error bars reflect 1 standard deviation.

While several pairwise platform comparisons have already been discussed, it is perhaps instructive to look at how three different platforms did in the identification and quantification of the one group of compounds that all three platforms measured: amino acids. Comparison of amino acid concentrations as measured by NMR, GC-MS and DFI MS/MS showed that the quantitative results are in relatively good agreement (Figure 4) A few exceptions are evident. For example, the NMR concentration of L-alanine is considerably higher than the GC-MS value. This may be due to the short GC retention time of L-alanine (∼7 min), which overlaps with non-specified ionized fragments and so an accurate quantification is impeded. L-Leucine and L-isoleucine cannot be distinguished with the Biocrates kit and therefore their concentrations have been combined from NMR and GC-MS measurements in order to make them comparable with the Biocrates result.

The considerably higher concentration of L-glutamic acid and lower concentration of L-glutamine reported by GC-MS relative to NMR and DFI MS/MS may be due to the hydrolysis of L-glutamine to L-glutamic acid [92] or the conversion of L-glutamine to pyroglutamic during derivatization [93]. It has been reported that the distinction of L-glutamine and pyroglutamic acid with GC-MS is very difficult, while the identification of L-glutamic acid and pyroglutamic acid can be complicated [92]. Moreover, it has been noted that L-cystine can be converted to L-cysteine during derivatization, while L-cysteine might be oxidized to L-cystine during prolonged storage of the standard solution [93]. Therefore, L-cystine and L-cysteine determinations have unavoidable errors unless they are converted prior to quantification. Finally, L-arginine and ornithine could not be accurately quantified by GC-MS, because firstly ornithine coelutes with citric acid (16.4 min) and secondly because L-arginine is converted to ornithine during derivatization [94].

While side-by-side platform comparisons for the quantification of specific metabolites are quite informative, it is also instructive to compare the different platforms on the basis of their level of metabolitle coverage. Given that the known, quantifiable serum metabolome consists of 4229 known and probable metabolites (665 literature derived metabolites and 3564 experimentally derived or predicted), we can calculate that NMR is able to measure ∼1.2% (49/4229) of the human serum metabolome, GC-MS is able to measure 2.13% (90/4229), ESI-MS/MS (lipid mediator profiling) is able to measure 2.3% (96/4229), TLC/GC-FID-MS (general lipidomics) is able to measure 79.9% (3381/4229) while DFI MS/MS is able to measure 3.3% (139/4229) of the serum metabolome. When combined the five methods are able to obtain data on 84% of the serum metabolome (3564/4229). It is important to emphasize that not all of the experimental approaches used in this study were “global” in their intent, meaning that the detection and quantification of these metabolites was not targeted. In particular, the DFI MS/MS and lipid mediator profiling methods were highly targeted, while the lipidomics method was generally targeted to lipids and fatty acids. Likewise, it is also important to emphasize that not all possible metabolomics platforms or technologies were assessed in this study nor were some of the latest metabolomics technologies. The use of more sophisticated or targeted detection and separation protocols (immunodetection, chemical derivitization, etc.) along with the use of higher-end instruments (GC-TOF, FT-MS, Orbitraps) would likely have led to the experimental detection of more compounds. However for this study, we wanted to address the question of how well a cross-section of commonly accessible metabolomic methods or platforms could perform in identifying and quantifying metabolites in serum.

Given the rich lipid character of serum, lipidomic methods including lipid mediator profiling, DFI MS/MS and general lipidomics methods such as TLC/GC-FID-MS appear to be the most suitable methods for serum characterization – both in terms of their breadth of coverage and their amenability to quantification. In fact, it has been recently postulated that the theoretical number of distinct lipid isoforms in the human body may approach 200,000 [95]. Even though the identification and quantification of such a vast number of lipids is currently not possible, new advances in MS-based technology are expanding the number and types of lipids that can be analyzed [95]. Generally among glycerophospholipids and glycerolipids, the determination of the length of each fatty acyl moiety at the sn-1 and sn-2 positions of the glycerol backbone and the total number of the double bonds in each lipid can be unambiguously determined [95], [96], [97]. However, the determination of the exact position of the double bond and the relative number of the positional isomers poses a major challenge in lipidomics [95], [98]. Other challenges also exist for this field including: 1) the observation that different classes of lipids may significantly affect instrument response of [99]; 2) the different patterns that identical lipid species may show when analyzed by various types of mass spectrometers or by the same mass spectrometer in different experimental modes [100] and 3) the limited availability of internal standards with the exact structure of the lipids of interest [101]. Even with these limitations, it is still clear that targeted lipid analysis of serum will always yield an enormous abundance or metabolites.

While NMR and GC-MS do provide information on many water-soluble metabolites, we believe that these methods are still insufficiently sensitive to compensate for their lack of coverage. Overall, it appears that LC-MS or DI-MS methods may be the best choice for serum metabolomic studies, despite their bias against hydrophilic metabolites. Interestingly, the use of hydrophobic enrichment tags (similar in concept to trimethylsilation in GC-MS) using p-chlorophenylalanine mediated chemical labeling [102], dimethyl isotopic labeling [103] or dansyl chloride labeling [104] has been shown to confer enhanced LC retention and improved MS-detection of hydrophilic metabolites. Preliminary data (Liang Li, personal communication) suggests this chemo-selective tagging approach could lead to detection and relative quantification by LC-FTMS of perhaps 400 water-soluble metabolites in the serum metabolome.

To summarize, this particular study of the human serum metabolome was designed to address four key questions: (1) what compounds can be or have ever been identified in human serum? (2) What are the concentration ranges for these metabolites? (3) What portion of the human serum metabolome can be routinely identified and/or quantified using conventional, untargeted metabolomics methods? (4) What analytical methods (NMR, GC–MS, GC-FID, LC–MS) are best suited for comprehensively characterizing the human serum metabolome? The answers to the first two questions have already been given and the information is contained in the human serum metabolome database (SMDB – http://www.serummetabolome.ca). With respect to the third question, given that the known, quantifiable serum metabolome consists of 4229 confirmed and probable metabolites, we observe that NMR is able to measure ∼1.2% (49/4229) of the human serum metabolome, GC-MS is able to measure 2.13% (90/4229), ESI-MS/MS (lipid mediator profiling) is able to measure 2.3% (96/4229), TLC/GC-FID-MS (general lipidomics) is able to measure the concentration of 79.9% (3381/4229) while DFI MS/MS is able to access 3.3% (139/4229) of the serum metabolome. When combined the five methods are able to obtain data on 84% of the serum metabolome (3564/4229).

While clear differences do exist in the number and type of compounds detected by the technologies employed in this study, the intent was not to denigrate any technology, but simply to explore their limits (strengths and weaknesses) and to characterize the human serum metabolome with a cross-section of commonly available metabolomics tools or platforms. Indeed, this study suggests that comprehensive metabolite profiling of human serum requires multiple platforms and multiple methods as no single method can offer (nor likely will offer) complete metabolite coverage. Despite these caveats, it is fairly clear that non-targeted lipidomics (TLC/LC-GC-FID) using combinatorial lipid reconstruction appears to be the best method for getting the largest degree of metabolite coverage, even though many of the metabolites and concentrations generated through the CLR method would have to be called “probable” rather than confirmed. However, this approach is time-consuming, expensive, requires relatively large sample volumes and is focused on the lipid classes specifically, thus providing limited coverage of metabolic space. The use of targeted metabolite profiling approaches (such as lipid mediator profiling or the DI MS/MS kit), while not as comprehensive, shows good promise and exceptional sensitivity. Furthermore, both methods allow specific expansion into other regions of metabolic space. In particular, the low volume requirements (10–20 µL) and the high-throughput nature (80 samples/day) of kit-based technologies such as the Biocrates Absolute*IDQ* kit could make this approach particularly appealing to many labs. While NMR may be the most robust technology for quantitative metabolomics, the high volume requirements (>300 µL) and general lack of sensitivity (>1 µM) tend to make this approach somewhat limiting. The reduced sensitivity obviously means NMR-based approaches will tend to miss many low abundance serum metabolites (i.e. inflammatory or oxidation-status markers) of clinical interest. Overall, GC–MS appears to have similar or slightly better sensitivity than NMR spectroscopy, although quantification by GC-MS tends to be more difficult. It is notable that GC-MS volume requirements are often substantially less than NMR, making GC-MS a more powerful approach to doing metabolomics with volume-limited samples. Potentially, the use of GC–TOF instrument or a fast scanning quadrupole instrument would have yielded even more favorable results for our GC–MS studies.

Obviously, if time and resources permitted, we would have liked to assess other technologies and to study a much broader patient base. However, this study is not the “final” word on serum or blood metabolomics. Rather, it should be viewed as a starting point for future studies and future improvements in this field. Indeed, our primary objective for undertaking these studies and compiling this data was to help advance the fields of quantitative metabolomics, especially with regard to clinically important biofluids. Experimentally, our data should serve as a useful benchmark from which to compare other technologies and to assess coming methodological improvements in human serum characterization. From a clinical standpoint, we think the information contained in the human serum metabolome database (SMDB) should provide clinicians and clinical chemists a convenient, centralized resource from which to learn more about human serum and its unique biochemical functions.

## Methods

### Ethics Statement

All samples were collected in accordance with the ethical guidelines and written consent protocols mandated by the University of Alberta, the University of British Columbia (UBC) and the Pennington Biomedical Research Centre (PBRC). All three institutional review boards approved the collection of serum for comprehensive metabolite characterization. All patients and all control individuals were approached using approved ethical guidelines and those who agreed to participate in this study, were required to sign consent forms. Patients could refuse entry, discontinue participation, or withdraw from the study at any time without prejudice to further treatment or management. All participants provided written consent.

### Sample Collection and Preparation

Four different sets of blood or serum samples were collected for our experimental studies (Table 1). A set of 54 adult serum samples was collected specifically for quantitative NMR, untargeted GC-MS and targeted (DFI) MS/MS studies, and a second set of 3 samples was collected and analyzed using quantitative lipidomics assays (TLC-methyl-esterification-GC-MS) developed by Lipomics Technologies Inc. (now Tethys Inc., West Sacramento, CA). The small number of samples used for the lipidomic assays were dictated by the substantial time and cost associated with these targeted quantitative studies. A third set of plasma samples was collected from 70 healthy subjects for the determination of nonesterified lipids and lipid mediators, including oxylipins and endocannabionids, by targeted GC-MS and UPLC-MS/MS. A fourth set comprised of 3 technical replicates of one adult serum sample, was collected and analyzed for oxylipins by UPLC-ESI-MS/MS in an independent analysis.

**Table 1:** Summary of sample collection and analysis methods.

Number of Samples	Sample Source	Number of samples analyzed by different methods						
		NMR	Untargeted GC-MS	Targeted GC-MS (oxylipins, endo-cannabionids)	Targeted (DFI) MS/MS	UPLC-MS/MS (oxylipins, endocannabi-onids)	Quantitative Lipidomics (TLC-methyl-esterfication-GC-MS)	Analysis Location
75 (54 Patients, 21 Controls	James Hogg iCAPTURE Centre for Cardiovascular and Pulmonary Research and the NCE CECR Centre of Excellence for Prevention of Organ Failure (PROOF Centre), Vancouever BC, Canada	75	7 controls	-	21 controls	-	-	Edmonton AB, Canada
3	Clinical Laboratory Medicine, University of Alberta, Edmonton AB, Canada	-	-	-	-	-	3	West Sacramento CA, USA; Edmonton AB, Canada
70	Pennington Biomedical Research Center, Baton Rouge LA, USA	-	-	70	-	70	-	Davis CA, USA
1	Clinical Laboratory Medicine, University of Alberta, Edmonton AB, Canada	-	-		-	1 (three technical replicates for oxylipin analysis)	-	Davis CA, USA

**Table 2:** Chemical classes in the Serum Metabolome Database.

Compound class	Number	Compound class	Number
Acyl glycines	**10**	Indoles and indole derivatives	**12**
Acyl phosphates	**10**	Inorganic ions and gases	**20**
Alcohol phosphates	**2**	Keto acids	**8**
Alcohols and polyols	**40**	Ketones	**6**
Aldehydes	**3**	Leukotrienes	**8**
Alkanes and alkenes	**10**	Lipoamides and derivatives	**0**
Amino acid phosphates	**1**	Minerals and elements	**40**
Amino acids	**114**	Miscellaneous	**77**
Amino alcohols	**14**	Nucleosides	**24**
Amino ketones	**14**	Nucleotides	**24**
Aromatic acids	**22**	Peptides	**21**
Bile acids	**19**	Phospholipids	**2177**
Biotin and derivatives	**2**	Polyamines	**11**
Carbohydrates	**35**	Polyphenols	**22**
Carnitines	**22**	Porphyrins	**6**
Catecholamines and derivatives	**21**	Prostanoids	**23**
Cobalamin derivatives	**4**	Pterins	**14**
Coenzyme A derivatives	**1**	Purines and purine derivatives	**11**
Cyclic amines	**9**	Pyridoxals and derivatives	**7**
Dicarboxylic acids	**17**	Pyrimidines and pyrimidine derivatives	**2**
Fatty acids	**65**	Quinones and derivatives	**3**
Glucuronides	**8**	Retinoids	**11**
Glycerolipids	**1070**	Sphingolipids	**3**
Glycolipids	**15**	Steroids and steroid derivatives	**109**
Hydroxy acids	**129**	Sugar phosphates	**9**
		Tricarboxylic acids	**2**

**Table 3:** Concentrations (Mean ± stdev) and % occurrence of serum metabolites as determined by NMR.

Compound Name	Healthy Subject	Heart Transplant	Literature Value
	µM; (% Occurrence)	µM; (% Occurrence)	µM; (Range)
2-Hydroxybutyric acid	31.3±7.8; (73%)	24.3±14.5; (92%)	54; (8–80)
Alpha-ketoisovaleric acid	ND	10.7±5.5; (40%)	NA
3-Hydroxybutyric acid	76.9±66.3; (80%)	35.1±33.9; (96%)	60.0±20.0
Acetaminophen	ND	33.5±22.3; (8%)	NA
Acetic acid	41.9±15.1; (100%)	42.2±17.3; (100%)	30; (22–40)
Acetoacetic acid	40.6±36.5; (33%)	27.3±14.4; (25%)	21.0; (0.0–86.0)
Acetone	54.4±29.6; (86%)	13.2±5.5; (4%)	106; (35–170)
L-Alanine	427.2±84.4; (100%)	340±126.2; (100%)	333; (259–407)
L-Arginine	113.6±14.6; (100%)	ND	111.6; (82.2–140.9)
L-Asparagine	82.4±7.3; (100%)	54.1±21.7; (42%)	41±10
L-Aspartic acid	20.9±6.1; (100%)	ND	21.0+/−5.0
Betaine	72±22.4; (100%)	42.1±19.3; (100%)	82; (20–144)
L-Carnitine	45.7±11.6; (100%)	41.7±23.9; (100%)	43; (26–79)
Choline	14.5±5.3; (90%)	9.7±4.5; (92%)	10.6±1.9
Citric acid	114.2±27; (100%)	80.2±44.9; (100%)	190; (30–400)
Creatine	36.7±28.3; (100%)	33.8±37.7; (100%)	54.8±21.0
Creatinine	86.6±18.8; (100%)	86.9±44.5; (100%)	74.1±10.9
L-Cysteine	33.5±10.3; (100%)	ND	52.0; (41.0–63.0)
L-Cystine	62.9±27.8; (100%)	ND	109.0±24.0
Ethanol	ND	40.2±12.1; (13%)	NA
Formic acid	32.8±13.3; (48%)	19.8±6.8; (60%)	121.7±97.8
D-Glucose	4971.3±372.8; (100%)	3743±1272.9; (100%)	5400; (4700–6100)
L-Glutamic acid	97.4±13.2; (100%)	72±36.9; (40%)	21.0–150.0
L-Glutamine	510.4±118.2; (100%)	376.8±114.3; (100%)	586; (502–670)
Glycerol	431.6±100.4; (100%)	133.9±87.8; (100%)	82; (27–137)
Glycine	325.4±126.8; (100%)	234.9±181.1; (100%)	230; (178–282)
L-Histidine	131.2±37.3; (100%)	46.1±17.5; (100%)	82; (72–92)
Hypoxanthine	34.2±10.3; (24%)	52.3±*; (2%)	8.1; (5.3–11.0)
Isobutyric acid	ND	8.4±1.9; (11%)	NA
L-Isoleucine	60.7±18.6; (100%)	44.6±21.5; (100%)	62; (48–76)
Isopropyl alcohol	83.3±132.8; (48%)	16.5±22.5; (45%)	Not available
L-Lactic acid	1489.4±371.2; (100%)	1401.2±692.1; (100%)	1510; (740–2400)
L-Leucine	98.7±11.5; (100%)	74.8±34.3; (100%)	123; (98–148)
L-Lysine	178.6±58.2; (100%)	128.2±55.3; (100%)	183.0±34.0
Malonic acid	13.5±1.2; (14%)	105.7±95.8; (9%)	15.0±0.6
Methanol	77.4±16.3; (100%)	81.5±55.2; (94%)	47.2±10.3
L-Methionine	29.8±6.3; (33%)	17.3±9.5; (66%)	30; (22–38)
Methylmalonic acid	ND	11.2±*; (2%)	NA
L-Ornithine	66.9±15.3; (100%)	65.4±30.4; (100%)	55; (39–71)
L-Phenylalanine	78.1±20.5; (100%)	44.8±21; (94%)	65.0±9.0
L-Proline	198.3±64.8; (100%)	159.9±86.3; (100%)	239.0±70.0
Propylene glycol	22.3±3.3; (100%)	36.3±19.9; (62%)	2; (0–5)
Pyruvic acid	34.5±25.2; (81%)	50.2±40; (87%)	64; (22–258)
L-Serine	159.8±26.6; (100%)	ND	137.0±35.0
L-Threonine	127.7±41; (100%)	83.4±47.8; (96%)	140; (107–173)
L-Tryptophan	54.5±9.7; (100%)	ND	48.7±11.6
L-Tyrosine	54.5±9.7; (100%)	57.2±24.4; (100%)	100; (55–147)
Urea	6074.6±2154.2; (100%)	3309.9±1844; (100%)	6500; (4000–9000)
L-Valine	212.3±61.3; (100%)	144.2±61.4; (100%)	233; (190–276)
Xanthine	ND	51.2±*; (2%)	NA

*- only observed in one sample.

**Table 4:** List of 74 metabolites identified in human serum polar and lipid extracts.

Amino acids	Organic acids	Lipids	Misc
Glycine	2-aminobutyric acid	Arachidonic acid	D-Fructose
L-Alanine	Alpha-Hydroxyisobutyric acid	Cholesterol	D-Galactopyranose
L-Asparagine	2-Methylbutanoic acid	Capric acid	D-Galactose
L-Aspartic acid	3-Hydroxybutyric acid	Dodecanoic acid	Glucitol
L-Cysteine	4-Hydroxybutyric acid	Arachidic acid	D-Glucose
L-Cystine	Aminomalonic acid	Heptadecanoic acid	Glycerol
L-Glutamic acid	Benzoic acid	Linoleic acid	D-Glucopyranose
L-Glutamine	Citric acid	Oleic acid	Hydroxyproline
L-Histidine	Erythronic acid	Palmitelaidic acid	D-Maltose
L-Isoleucine	Fumaric acid	Palmitic acid	Myo-inositol
L-Leucine	Gluconic acid	Stearic acid	Acetylglycine
L-Lysine	Glyceric acid	Myristic acid	N-Acetyl-L-Lysine
L-Methionine	Isobutyric acid		Acetaminophen
L-Ornithine	Tartaric acid		Phosphoric acid
L-Phenylalanine	L-Lactic acid		Ribitol
L-Proline	Malonic acid		Salicylic acid
L-Serine	Methylmaleic acid		Urea
L-Threonine	Methylmalonic acid		D-Xylitol
L-Tryptophan	Nicotinic acid		
L-Tyrosine	Oxalic acid		
L-Valine	Pyroglutamic acid		
	Succinic acid		
	Uric acid		

**Table 5 pone-0016957-t005:** Non-esterified fatty acid concentrations (µM) detected by GC-MS in human plasma.

Compound Name	Class	HMDB ID	Common Abbreviaton	Pennington Plasma (n = 70)
Dodecanoic acid	SAT	HMDB00638	C12:0	1.47±0.68
Myristic acid	SAT	HMDB00806	C14:0	7.16±3
Pentadecanoate	SAT	HMDB00826	C15:0	1.34±0.91
Palmitic acid	SAT	HMDB00220	C16:0	122±48
Heptadecanoic acid	SAT	HMDB02259	C17:0	1.89±0.92
Stearic acid	SAT	HMDB00827	C18:0	48.8±21
Palmitelaidic acid	MUFA	HMDB12328	C16:1n7t	1.97±1.4
Palmitoleic acid	MUFA	HMDB03229	C16:1n7	9.66±6.8
Vaccenic acid	MUFA	HMDB03231	C18:1n7	10.7±5
Oleic acid	MUFA	HMDB00207	C18:1n9	122±56
Nonadeca-10(Z)-enoic acid	MUFA	HMDB13622	C19:1n9	0.646±0.37
Eicosenoic acid	MUFA	HMDB02231	C20:1n9	0.663±0.59
Linoleic acid	PUFA	HMDB00673	C18:2n6	83.8±38
Gamma-Linolenic acid	PUFA	HMDB03073	C18:3n6	1.08±1.5
Bovinic acid	PUFA	HMDB03797	C18:2(9c/t,11t)-CLA	2.03±1.3
Alpha- Linolenic acid	PUFA	HMDB01388	C18:3n3	5.11±3.8
Mead acid	PUFA	HMDB10378	C20:3n9	0.987±0.45
Dihomo-gamma-linolenic acid	PUFA	HMDB02925	C20:3n6	3.61±2.1
Arachidonic acid	PUFA	HMDB01043	C20:4n6	14±12
Adrenic acid	PUFA	HMDB02226	C22:4n6	1.01±0.48
-4,7,10,13,16-Docosapentaenoic acid	PUFA	HMDB13123	C22:5n6	0.953±0.51
Stearidonic acid	PUFA	HMDB06547	C18:4n3	0.408±0.4
Timnodonic acid; EPA	PUFA	HMDB01999	C20:5n3	1.09±0.72
Clupanodonic acid; DPA	PUFA	HMDB06528	C22:5n3	0.993±0.46
Cervonic acid; DHA	PUFA	HMDB02183	C22:6n3	4.66±3.3

**Table 6:** Concentrations of metabolites in healthy serum performed by GC-MS.

Metabolites	Mean (µM)	Literature values (µM)
Oxalic acid	22.2	9.2±2.7
Acetylglycine	69.7	109.4±85.6
Myo-inositol	17.1	23.0±8.0
Uric acid	494.2	302±60
Succinic acid	23.5	16.0 (0.0–32.0)
Alpha-Hydroxyisobutyric acid	8.2	7.0 (0.0–9.0)
Ribitol/D-Xylitol	<5	0.46 (0.38–0.55)
Erythronic acid	<5	2.5 (0.0–5.0)
Lauric (Dodecanoic) acid	9.1	12.0 (2.0–37.0)
Phosphoric acid	820.4	1100 (810–1450)
Myristic (Tetradecanoic) acid	9.3	15.4±4.0
Gluconic acid	<5	NA
D-Maltose/L-Arabinose	<5	2.5 (0.0–5.0)
Glyceric acid	<5	2.5 (0.0–5.0)

**Table 7:** Omega-6 oxylipins (nM) detected by UPLC (−)ESI-MS/MS in human plasma.

Parent Lipid	Class^a^	HMDB ID	Common Abbreviaton	HM Replicate Plasma (n = 3)	Pennington Plasma (n = 70)
C20:4n6	R-OH	HMDB05998	20-HETE	1.77±0.43	0.917±0.58
C20:4n6	R-OH	HMDB03876	15-HETE	1.8±0.098	2.04±1.2
C20:4n6	R-OH	HMDB04682	11-HETE	0.425±0.0095	0.401±0.36
C20:4n6	R-OH	HMDB06111	12-HETE	6.42±0.74	3.95±3.3
C20:4n6	R-OH	HMDB10222	9-HETE	0.304±0.072	0.166±0.16
C20:4n6	R-OH	HMDB04679	8-HETE	2.09±0.16	0.536±0.4
C20:4n6	R-OH	HMDB11134	5-HETE	0.901±0.029	1.02±0.79
C20:4n6	R = O	HMDB10210	15-KETE	0.749±0.08	0.682±0.76
C20:4n6	R = O	HMDB13633	12-KETE	<0.1	<0.1
C20:4n6	R = O	HMDB10217	5-KETE	0.136±0.018	0.145±0.12
C20:4n6	R-OOH	HMDB04244	15-HPETE	NA	1.06±0.41
C20:4n6	R-OOH	HMDB04243	12-HPETE	NA	1.45±2.3
C20:4n6	Diol	HMDB04385	Lipoxin A4	<0.07	<0.07
C20:4n6	Diol	HMDB01085	LTB4	0.0968±0.0062	<0.1
C20:4n6	Diol	HMDB05087	6-trans-LTB4	0.223±0.042	<0.1
C20:4n6	Triol	HMDB01509	20-hydroxy-LTB4	NA	<0.1
C20:4n6	Diol	HMDB06059	20-carboxy-LTB4	NA	<1
C20:4n6	Diol	HMDB10216	5,15-DiHETE	0.247±0.02	<0.07
C20:4n6	Diol	HMDB10219	8,15-DiHETE	<0.1	<0.1
C20:4n6	Diol	HMDB02265	14,15-DiHETrE	0.714±0.031	0.603±0.18
C20:4n6	Diol	HMDB02314	11,12-DiHETrE	0.779±0.037	0.566±0.2
C20:4n6	Diol	HMDB02311	8,9-DiHETrE	0.294±0.056	0.244±0.078
C20:4n6	Diol	HMDB02343	5,6-DiHETrE	0.264±0.025	0.189±0.092
C20:4n6	Epox	HMDB04693	14(15)-EpETrE	1.77±0.05	0.442±0.59
C20:4n6	Epox	HMDB10409	11(12)-EpETrE	0.303±0.028	1.02±1.4
C20:4n6	Epox	HMDB02232	8(9)-EpETrE	<0.2	0.627±0.71
C20:4n6	Epox	HMDB04688	Hepoxilin A3	NA	0.114±0.087
C20:4n6	LT	HMDB02200	LTE4	NA	<0.6
C20:4n6	TX	HMDB03252	TXB2	0.865±0.18	0.919±1.6
C20:4n6	PG	HMDB02886	6-keto-PGF1a	0.359±0.023	0.0607±0.028
C20:4n6	PG	HMDB01139	PGF2a	0.33±0.018	0.248±0.13
C20:4n6	PG	HMDB01220	PGE2	0.0967±0.012	0.172±0.13
C20:4n6	PG	HMDB01403	PGD2	*0.0726*±*0.0058*	<0.1
C20:4n6	PG	HMDB02710	PGJ2	<0.3	<0.3
C20:4n6	PG	HMDB04236	PGB2	*0.519*±*0.096*	<0.7
C20:4n6	PG	HMDB04238	Delta-12-PGJ2	<0.3	<0.3
C20:4n6	PG	HMDB05079	15-deoxy PGJ2	0.206±0.011	<0.3
C20:4n6	Triol	HMDB04684	11,12,15-TriHETrE	<0.1	<0.1
C20:3n6	R-OH	HMDB05045	15-HETrE	0.437±0.028	0.732±0.45
C20:3n6	PG	HMDB01442	PGE1	<0.1	<0.1

a Class: R-OH - hydroxy fatty acid; R = O - keto fatty acid; Diol - dihydroxy fatty acid; Triol - trihydroxy fatty acid; Epox - epoxy fatty acid; LT - leukotriene; PG - prostaglandin.

**Table 8:** Omega-3 oxylipins (nM) detected by UPLC (−)ESI-MS/MS in human plasma.

Parent Lipid	Class^a^	HMDB ID	Common Abbreviaton	HM Replicate Plasma (n = 3)	Pennington Plasma (n = 70)
C20:5n3	R-OH	HMDB10209	15-HEPE	0.28±0.042	1.63±1.6
C20:5n3	R-OH	HMDB10202	12-HEPE	3.19±0.35	0.195±0.11
C20:5n3	R-OH	HMDB05081	5-HEPE	1.15±0.14	0.228±0.091
C20:5n3	Diol	HMDB10211	17,18-DiHETE	14.4±1.1	2.08±0.85
C20:5n3	Diol	HMDB10204	14,15-DiHETE	ND	0.304±0.1
C20:5n3	Epox	HMDB10212	17,18-EpETE	ND	0.0733±0.095
C20:5n3	Epox	HMDB10205	14,15-EpETE	0.119±0.029	<0.1
C20:5n3	LT	HMDB05073	LTB5	0.079±0.0056	<0.1
C20:5n3	PG	HMDB02664	PGE3	ND	<0.1
C20:5n3	Triol	HMDB10410	Resolvin E1	1.00±0.23	0.521±0.98
C22:6n3	Epox	HMDB13620	19(20)-EpDoPE	ND	<0.1
C22:6n3	Epox	HMDB13621	16(17)-EpDoPE	ND	0.368±0.43
C22:6n3	Diol	HMDB10214	19,20-DiHDoPE	ND	0.805±0.42
C22:6n3	R-OH	HMDB10213	17-HDoHE	ND	0.773±0.64
C22:6n3	Triol	HMDB03733	Resolvin D1	ND	0.0454±0.027

a Class: R-OH - hydroxy fatty acid; R = O - keto fatty acid; Diol - dihydroxy fatty acid; Triol - trihydroxy fatty acid; Epox - epoxy fatty acid; LT - leukotriene; PG - prostaglandin.

**Table 9:** Octadecanoid oxylipins (nM) detected by UPLC (−)ESI-MS/MS in human plasma.

Parent Lipid	Class*^a^*	HMDB ID	Common Abbreviaton	HM Replicate Plasma (n = 3)	Pennington Plasma (n = 70)
C18:2n6	Diol	HMDB04705	12,13-DiHOME	7.69±0.59	5.82±3
C18:2n6	Diol	HMDB04704	9,10-DiHOME	60.5±3.8	29.7±11
C18:2n6	Epox	HMDB04702	12(13)-EpOME	4.88±0.34	7.21±8.8
C18:2n6	Epox	HMDB04701	9(10)-EpOME	2.17±0.23	5.47±7.4
C18:2n6	R-OH	HMDB04667	13-HODE	47.3±0.53	58.2±28
C18:2n6	R-OH	HMDB10223	9-HODE	11.7±0.23	11±6.1
C18:2n6	R-OOH	HMDB03871	13-HpODE	ND	6.01±5.5
C18:2n6	R-OOH	HMDB06940	9-HpODE	ND	5.14±3.8
C18:2n6	Epox,R = O	HMDB13623	12(13)Ep-9-KODE	3.02±0.27	3.96±2.4
C18:2n6	R = O	HMDB04668	13-KODE	4.82±0.68	1.7±1.2
C18:2n6	R = O	HMDB04669	9-KODE	2.41±0.29	5.3±2.7
C18:2n6	Triol	HMDB04708	9,12,13-TriHOME	0.827±0.21	4.11±2.2
C18:2n6	Triol	HMDB04710	9,10,13-TriHOME	0.513±0.083	1.16±0.64
C18:3n3	Diol	HMDB10208	15,16-DiHODE	14.5±1	5.93±2.4
C18:3n3	Diol	HMDB10201	12,13-DiHODE	<0.2	0.219±0.12
C18:3n3	Diol	HMDB10221	9,10-DiHODE	2.36±0.15	0.114±0.085
C18:3n3	Epox	HMDB10206	15(16)-EpODE	3.27±0.23	2.77±2.1
C18:3n3	Epox	HMDB10200	12(13)-EpODE	0.416±0.08	0.468±0.67
C18:3n3	Epox	HMDB10220	9(10)-EpODE	2.08±0.075	1.65±2.3
C18:3n3	R-OH	HMDB10203	13-HOTE	1.9±0.21	1.11±0.74
C18:3n3	R-OH	HMDB10224	9-HOTE	1.98±0.12	1.19±0.91

Class: R-OH - hydroxy fatty acid; R = O - keto fatty acid; Diol - dihydroxy fatty acid; Triol - trihydroxy fatty acid; Epox - epoxy fatty acid; LT - leukotriene; PG - prostaglandin.

**Table 10:** Acyl- ethanolamide, -glycerols, and -glycines concentrations (nM) detected by UPLC (+)ESI-MS/MS in human plasma.

Parent Lipid	Class	HMDB ID	Common Abbreviaton	Pennington Plasma (n = 70)
C16:0	Ethanolamide	HMDB02100	PEA	25.1±12
C18:0	Ethanolamide	HMDB13078	SEA	15±11
C18:1n9	Ethanolamide	HMDB02088	OEA	46.8±34
C18:2n6	Ethanolamide	HMDB12252	LEA	13.7±6.5
C18:3n3	Ethanolamide	HMDB13624	Alpha-LEA	0.118±0.069
C20:3n6	Ethanolamide	HMDB13625	DGLA EA	1.01±0.48
C20:4n6	Ethanolamide	HMDB04080	AEA	3.12±1.2
C22:4n6	Ethanolamide	HMDB13626	DEA	1.63±0.78
C22:6n3	Ethanolamide	HMDB13627	DHEA	0.401±0.22
PGF2a	Ethanolamide	HMDB13628	PGF2a EA	0.0173±0.015
PGD2	Ethanolamide	HMDB13629	PGD2 EA	0.161±0.032
20-HETE	Ethanolamide	HMDB13630	20-HETE EA	0.0208±0.013
C18:1n9	1-Acyl Glycerol	HMDB11567	1-OG	170±170
C18:2n6	1-Acyl Glycerol	HMDB11568	1-LG	37.6±36
C20:4n6	1-Acyl Glycerol	HMDB11578	1-AG	4.71±4.5
C18:1n9	2-Acyl Glycerol	HMDB11537	2-OG	166±130
C18:2n6	2-Acyl Glycerol	HMDB11538	2-LG	146±97
C20:4n6	2-Acyl Glycerol	HMDB04666	2-AG	7.8±4.6
C18:1n9	N-Acyl Glycine	HMDB13631	NO-Gly	21±23
C20:4n6	N-Acyl Glycine	HMDB05096	NA-Gly	1.09±0.73

**Table 11:** Concentrations (µM) of cholesterol esters, free fatty acids and lysophospatidylcholines as quantified by TLC/GC-FID.

	Cholesterol esters (CEs)		Free Fatty Acids (FFAs)		Lysopsosphatidylcholines (LysoPCs)	
Lipid Class	Mean	SD	Mean	SD	Mean	SD
**C14:0**	97.04	59.91	15.46	4.02	4.23	1.67
**C15:0**	ND	ND	2.69	0.51	1.76	0.85
**C16:0**	405.46	56.51	66.01	9.88	106.60	16.73
**C18:0**	37.50	2.82	41.12	5.52	47.54	8.38
**C20:0**	1.18	0.12	0.87	0.09	0.69	0.37
**C22:0**	1.08	0.48	1.01	0.26	0.43	0.09
**C24:0**	0.91	0.55	0.93	0.19	0.70	0.35
**C14:1n5**	4.19	1.04	2.02	0.73	0.21	0.08
**C16:1n7**	118.75	45.54	6.39	4.28	2.34	1.05
**C18:1n7**	44.23	3.82	2.55	1.31	3.66	0.14
**C18:1n9**	704.47	129.59	49.24	19.31	37.47	7.73
**C20:1n9**	0.11	NA	1.50	1.21	0.53	0.17
**C20:3n9**	2.80	1.49	1.32	1.36	0.25	0.07
**C22:1n9**	1.86	2.43	1.26	1.59	0.57	0.26
**C24:1n9**	ND	ND	0.90	0.97	0.73	0.56
**C18:2n6**	1506.38	204.89	14.73	4.33	52.75	6.48
**C18:3n6**	23.66	1.58	0.31	0.20	0.23	0.10
**C20:2n6**	4.29	4.02	0.42	0.16	0.71	0.25
**C20:3n6**	18.71	5.94	0.42	0.21	2.75	0.29
**C20:4n6**	195.48	21.36	5.26	2.07	8.39	0.98
**C22:2n6**	1.06	NA	0.42	0.07	0.10	0.11
**C22:4n6**	ND	ND	ND	ND	0.13	NA
**C22:5n6**	3.28	3.46	0.14	0.09	0.11	0.02
**C18:3n3**	23.60	0.13	1.98	1.22	1.24	0.54
**C18:4n3**	ND	ND	ND	ND	0.29	0.32
**C20:4n3**	2.47	1.96	0.01	NA	0.26	0.14
**C20:5n3**	39.07	11.07	0.40	0.07	1.61	0.16
**C22:5n3**	3.22	3.67	0.39	0.23	0.74	0.22
**C22:6n3**	21.93	6.64	1.78	0.80	2.78	0.29
**dm16:0**	ND	ND	ND	ND	0.93	0.11
**dm18:0**	ND	ND	ND	ND	0.07	NA
**dm18:1n9**	ND	ND	ND	ND	0.13	NA

**Table 12:** Concentrations of acylcarnitines and amino acids (µM) in healthy serum by DFI MS/MS (Biocrates kit).

Acylcarnitines			Amino acids		
	Mean	SD		Mean	SD
**DL-carnitine**	29.738	7.547	**L-Arginine**	129.5	30.0
**Decanoylcarnitine**	0.260	0.111	**L-Glutamine**	492.6	93.6
**Decenoylcarnitine**	0.171	0.041	**Glycine**	329.9	105.6
**Decadienylcarnitine**	0.061	0.029	**L-Histidine**	143.1	27.3
**Dodecanoylcarnitine**	0.103	0.030	**L-Isoleucine+L-Leucine**	227.4	63.5
**Tetradecanoylcarnitine**	0.043	0.007	**L-Methionine**	33.4	9.0
**Tetradecenoylcarnitine**	0.063	0.028	**L-Ornithine**	93.8	41.3
**Tetradecadienylcarnitine**	0.028	0.013	**L-Pheylalanine**	85.2	23.0
**Hexadecanoylcarnitine**	0.072	0.019	**L-Proline**	177.5	38.6
**Hexadecenoylcarnitine**	0.029	0.005	**L-Serine**	173.2	51.3
**Hexadecadienylcarnitine**	0.012	0.002	**L-Threonine**	102.3	24.6
**Octadecanoylcarnitine**	0.035	0.010	**L-Tryptophan**	78.4	15.5
**Octadecenoylcarnitine**	0.108	0.036	**L-Tyrosine**	143.0	35.3
**Octadecadienylcarnitine**	0.035	0.013	**L-Valine**	266.3	61.0
**Acetyl-L-carnitine**	5.476	2.147			
**Propionyl-L-carnitine**	0.313	0.154	**Hexose**		
**Butyryl-L-carnitine**	0.262	0.158	**Hexose**	3767.6	607.0
**Hydroxybutyrylcarnitine**	0.106	0.010			
**Valeryl-L-carnitine**	0.142	0.063			
**Tiglyl-L-carnitine**	0.045	0.005			
**Glutaconyl-L-carnitine**	0.018	0.002			
**Octanoylcarnitine**	0.234	0.078			
**Octenoylcarnitine**	0.200	0.151			
**Nonaylcarnitine**	0.033	0.013			

**Table 13:** Concentrations of sphingomyelins and lysophosphatidylcholines (µM) in healthy serum by DFI MS/MS (Biocrates kit).

Sphingomyelins			Lysophosphatidylcholines		
	Mean	SD		Mean	SD
**SM (OH) C14:1**	5.92	1.63	**LysoPC a C14:0**	2.64	0.31
**SM (OH) C16:1**	3.70	0.90	**LysoPC a C16:0**	141	50
**SM (OH) C22:1**	15.6	3.7	**LysoPC a C16:1**	3.48	1.01
**SM (OH) C22:2**	12.89	2.88	**LysoPC a C17:0**	2.55	1.08
**SM (OH) C24:1**	2.56	0.66	**LysoPC a C18:0**	48.5	20.2
**SM C16:0**	100.5	18.6	**LysoPC a C18:1**	31.5	10.4
**SM C16:1**	15.1	3.5	**LysoPC a C18:2**	30.33	10.3
**SM C18:0**	25.8	6.4	**LysoPC a C20:3**	2.65	0.68
**SM C18:1**	11.7	3.0	**LysoPC a C20:4**	6.13	2.55
**SM C20:2**	1.34	0.35	**LysoPC a C24:0**	0.19	NA
**SM C22:3**	16.4	7.9	**LysoPC a C28:0**	0.370	0.043
**SM C24:0**	30.5	7.6	**LysoPC a C28:1**	0.481	0.117
**SM C24:1**	82.7	14.1			
**SM C26:0**	0.440	0.111			
**SM C26:1**	0.850	0.167			

**a: acyl.**

**Table 14:** Comparison of lysophosphatidylcholines concentrations (µM) performed by LC-ESI-MS/MS and DFI MS/MS (Biocrates kit).

	MS/MS (Biocrates)		LC/GC-FID (Lipomics)		
	Mean	SD		Mean	SD
**LysoPC a C14:0**	2.64	0.31		4.23	1.67
**LysoPC a C16:0**	124.1	50.46		106.6	16.73
**LysoPC a C16:1**	3.6	1.12		2.34	1.05
**LysoPC a C17:0**	2.36	1.04		ND	ND
**LysoPC a C18:0**	40.77	20.55		47.54	8.38
**LysoPC a C18:1**	30.94	10.05	**LysoPC C18:1n7**	3.66	0.14
			**LysoPC C18:1n9**	37.47	7.73
			**LysoPC dm18:1n9**	0.13	NA
**LysoPC a C18:2**	32.98	13.31		52.75	6.48
**LysoPC a C20:3**	2.53	0.74	**LysoPC C20:3n6**	2.75	0.29
			**LysoPC C20:3n9**	0.25	0.07
**LysoPC a C20:4**	6.13	2.47	**LysoPC C20:4n6**	8.39	0.98
			**LysoPC C20:4n3**	0.26	0.14
**LysoPC a C24:0**	0.19	NA		0.70	0.35
**LysoPC a C28:0**	0.37	0.06		ND	ND
**LysoPC a C28:1**	0.48	0.1		ND	ND

For the untargeted NMR and GC-MS studies, we also explored temporal and disease-associated metabolite differences to get a better idea of the extent of the cross-sectional and longitudinal metabolite variability for NMR-detectable blood metabolites. Because of the close clinical monitoring and frequent blood sampling done of organ transplant patients, we chose to work with a small cohort of heart transplant patients enrolled as part of the Biomarkers in Transplantation study at the James Hogg iCAPTURE Centre at St. Paul's Hospital in Vancouver BC. As a result, for the NMR, GC-MS and MS-MS studies, serum samples were collected from 21 healthy adult individuals (aged 26–71) (mentioned above) and 9 heart transplant patients (aged 26–64). Six serum samples were collected for each of the heart transplant patients at various time points (before transplantation, then 2, 3, 4, 8, and 12 weeks after transplantation) for a total of 9×6 = 54 samples. The heart transplant patients included 6 males and 3 females.

Blood samples obtained from the transplant patients (54 samples) or controls (21 for NMR, GC-MS, MS-MS, 1 for LC/MS, 3 for GC-MS/lipidomics) were collected via standard overnight fasting, vein-puncture methods and stored in serum tubes (with clot promoter). Samples were subsequently spun down for 15 minutes at 2900×g at 4°C and the serum decanted into clean plastic cryogenic vials and frozen to −80°C within 2 hours to minimize any possible metabolite degradation. All serum samples were thawed on ice for approximately 2 hours before use.

For the targeted analysis of nonesterified fatty acids, oxylipins, acylamides, and monoacylglycerols in plasma, compiled results were obtained from a study designed to assess the roles of lipids and hormones in regulating blood pressure in subjects with varying amounts of body fat. Subjects were healthy adults, ages 35 to 65 (32 males and 37 females) with each gender group comprising approximately half African Americans and half Caucasians. The subject groups displayed a representative range of body habitus with a BMI of 29±5 kg/m^2^. The study took place in a clinical research unit at the Pennington Biomedical Research Center in Baton Rouge, LA. A standard diet was given to subjects to eat for four days, three days while they lived at home, and the fourth day in the clinical research unit. Blood samples were drawn in the morning after an overnight fast and before subjects arose from bed. Venous blood was drawn into a tube containing EDTA, immediately chilled on ice for transport, centrifuged within an hour, and the plasma decanted into storage tubes. Storage was at −80°C for several months before analysis.

### NMR Compound Identification and Quantification

Serum samples contain a substantial portion of large molecular weight proteins and lipoproteins, which affects the identification and quantification of small molecule metabolites by NMR spectroscopy. Consequently, we introduced a step in the protocol to remove serum proteins (deproteinization). There are several routes to serum deproteinization, including organic solvent (acetonitrile, methanol, isopropanol) precipitation, ultrafiltration [28], [44] as well as spectral manipulation methods such as diffusion editing [45]. While other researchers have found that ultrafiltration yields poor signal-to-noise ratios, we found that by using an ultrafiltration protocol similar to that described by Tiziani [46] and Weljie et al [47], we could obtain excellent spectra that yielded metabolite concentrations that closely matched known values measured using standard clinical chemistry techniques. Ultrafiltration also has other advantages: it is relatively quick, very reproducible, does not introduce unwanted solvent peaks and is “safe” in terms of avoiding unwanted side-reactions with biofluid metabolites. All ^1^H-NMR spectra were collected on a either a 500 MHz or 800 MHz Inova (Varian Inc., Palo Alto, CA) spectrometer using the first transient of the tnnoesy-presaturation pulse sequence. The resulting ^1^H-NMR spectra were processed and analyzed using the Chenomx NMR Suite Professional software package version 6.0 (Chenomx Inc., Edmonton, AB), as previously described [15]. Further details on the NMR sample preparation and NMR data acquisition are provided in File S1.

### GC-MS Compound Identification and Quantification

Seven of the 21 normal serum samples were chosen for GC-MS analysis and aliquots from these provided an additional “pooled normal” sample (the heart transplant serum samples were not analyzed). All samples were extracted separately to obtain separate pools of polar and lipophilic metabolites using different protocols. The polar extraction protocol was adapted from a previously reported method [42] used to deproteinize and solubilize polar metabolites. Lipophilic metabolites were obtained by extracting serum samples using a mixture of cold HPLC grade chloroform/methanol as previously described [48]. All samples were derivatized with MSTFA (N-Methyl-N-trifluoroacetamide) with 1% TMCS (trimethylchlorosilane) and the resulting extracts were separated and analyzed using an Agilent 5890 Series II GC-MS operating in electron impact (EI) ionization mode. Further details on the extraction, derivatization, separation and GC-MS data analysis are provided in File S1.

### Targeted Profiling of Lipids and Lipid Mediators

Low to moderate abundance lipids and lipid-derived mediators in blood plasma were quantified using targeted GC-MS and LC-MS/MS analyses. Non-esterified fatty acids were methylated and quantified using modifications of the extractive methylation procedure of Pace-Asiack [49]. The GC-MS analyses were performed on an Agilent 6890 GC 5973N MSD with a 30 m×0.25 mm id×0.25 µm DB-225ms column using splitless injections (see File S1 for further details).

Oxylipins [50], [51], [52], [53], [54], [55] acylethanolamides [56], [57], [58], monoacylglycerols [59], and N-acylglycines [60], [61], [62] are important classes of low to moderate abundance blood-borne lipophilic molecules with recognized regulatory functions in inflammation, blood pressure, satiety, gut motility, energy balance, and pain regulation [54], [55]. While the importance of circulating levels of some of these metabolites is debated, given their clinical importance, we believed that establishing their normal ranges in the circulating blood metabolome would be valuable. However, as a host of these agents are either involved in or affected by the clotting process, their values were determined in plasma (not serum) from blood collected in the presence of potassium EDTA. These benchmark values were established in an independent cohort of age (49±7 yr) and BMI (29±5 kg/m^2^) matched African American and Caucasian men and women (n = 70). The plasma samples were extracted by solid phase extraction (SPE) and analyzed by UPLC-MS/MS for non-esterified oxylipins, acylethanolamides, N-acylglycines, and monoacylglycerols using modifications of previously described protocols [63]. Analytes were separated using an Acquity UPLC (Waters Corp), followed by MS/MS analysis on an ABI 4000 Q-Trap (Applied Biosystems). Oxylipins were detected using negative mode electrospray ionization, while all other residues were detected in positive mode. Compounds were quantified with six point calibration curves using a ratio response to stable isotope surrogates, using the Analyst software package (Applied Biosystems). Additional details on the extraction, calibration and processing are available in File S1.

### TLC/GC-FID Lipid Identification and Quantification

Serum and plasma are particularly rich in lipids and lipoprotein particles. To identify and quantify common lipids in serum a total of three human serum samples were analyzed using the TrueMass® platform developed by Lipomics Technologies Inc. (West Sacramento,CA; now Tethys Bioscience, Inc.). This method can be used to identify and quantify distinct lipid classes including neutral lipids, such as cholesterol esters (CEs), free fatty acids (FFAs), triacylglycerols (TGs) and diacylglycerols (DGs) as well as phospholipids such as lysophosphatidylcholines (LysoPCs), phosphatidylcholines (PCs) and phosphatidyl-ethanolamines (PEs). The methods used by Lipomics Technologies Inc. are described in more detail in a number of patents (US Patent #10753289, WO/2003/005628, see also reference [64] and File S1).

The major fatty acid constituents detected by this technique include all C12 to C24 saturated and unsaturated chains (>25 fatty acids in total). Quantification of the lipids and fatty acids was achieved using defined fatty acid methyl ester standards. A computational method called Combinatorial Lipid Reconstruction (CLR) was used to “regenerate” the structures and estimate the most probable concentrations of the triacylglycerols, diacylglycerols and phospholipids with >1 fatty acid chain. CLR uses the fractional abundance of each fatty acid chain and the total abundance of a given lipid class to estimate most probable and upper limit concentrations of a given lipid (see File S1 for a detailed description of CLR).

### Direct Flow Injection MS/MS Compound Identification and Quantification

To assess the performance of direct flow injection (DFI) MS/MS methods in serum metabolomics and to determine the concentration ranges of a number of metabolites not measurable by other methods, we used the commercially available Absolute*IDQ* kit (Biocrates Life Sciences AG - Austria). This kit, in combination with an ABI 4000 Q-Trap (Applied Biosystems/MDS Sciex) mass spectrometer, can be used for the targeted identification and quantification of 160 different metabolites including amino acids, acylcarnitines, glycerophospholipids, and sphingolipids. The method involves derivatization and extraction of analytes from the biofluid of interest, along with selective mass-spectrometric detection and quantification via multiple reaction monitoring (MRM). Isotope-labeled internal standards are integrated into the kit plate filter to facilitate metabolite quantification (see File S1 for additional information).

### Literature Survey of Human Serum Metabolites

In addition to these experimental studies, a complete literature review of known metabolites and metabolite concentrations in human serum and plasma was also conducted. A number of standard clinical chemistry textbooks [22], [27], [65], [66] provided reference values and disease concentrations for approximately 100 commonly measured blood metabolites. To supplement these data, we employed several computational text-mining tools that were originally developed for the Human Metabolome Database and subsequently used in the determination of the human cerebrospinal fluid metabolome [15]. One of the more useful programs was the in-house text mining tool called PolySearch [67] (http://wishart.biology.ualberta.ca/polysearch/). This program was used to generate a hyperlinked list of abstracts and papers from PubMed containing relevant information about serum metabolites and their concentration data. Specifically, PolySearch compiled a ranked list of metabolites based on the frequency of word co-occurrence with the terms “serum”, “plasma” or “blood” in conjunction with words such as “concentration”, “identification”, “quantification”, “mM”, “nM” or “micromol”. The list of metabolites and metabolite synonyms was compiled from the HMDB [4], [5], KEGG [6], ChEBI [9] and PubChem [68]. PolySearch also extracted key sentences from the abstracts, then labeled and hyperlinked the metabolites mentioned in the text. PolySearch processed more than 19 million abstracts to yield more than 2000 “highly informative” papers or abstracts. From these abstracts and papers, our annotators manually extracted metabolite information (metabolite identities, concentrations, disease states, etc.) and entered the data into our laboratory information management system (LIMS) called MetboLIMS. The resulting list of literature-derived metabolites helped confirm the identity of many metabolites found in our experimental analyses. The literature-derived concentration values also simplified some of the searches for putative metabolite matches in our experimental studies (described above). In total we identified 665 metabolites and obtained nearly 1000 metabolite concentration values using these bibliomic or text mining approaches.

### Metabolite Validation and Verification

A common weakness to many metabolomic studies is the presumption that all named metabolites are equally valid and fully verified. This is often not the case. For instance if a metabolite has been identified only through a parent ion mass match to a database compound, its identity should only be considered very tentative. On the other hand if a compound has been identified through an exact match to all 24 of its characteristic NMR peaks, or through matching the retention time, parent ion mass and mass fragment patterns of a known standard then obviously this compound's identity is on much more solid ground. In a study as broad and complex as this one, it is important to understand that some metabolites have only been tentatively identified while others have been fully confirmed or validated. Among the experimentally identified compounds reported here, authentic standards, exact NMR spectral matches or spike-in experiments were used to confirm the identity of almost all the compounds in our NMR, DFI MS/MS, oxylipin and lipidomics studies (see File S1 for additional details). Therefore most of these compounds should be considered as “confirmed”. For the CLR assembled lipids, all the component parts (head groups and acyl chains) were positively identified and verified using authentic standards, but the intact lipid could not be verified. Consequently the triacylglycerols, diacylglycerols and phospholipids reported here should be considered as “probable”. For the GC-MS studies some compound spiking was performed, but not every compound was validated with authentic standards. As a rule if a compound had an AMDIS match factor of >60% and a probability score >20% as well as a matching retention index to a known compound it was considered “probable”. If that compound had been previously identified by another method and if the concentration matched closely with a previously reported value, it was considered “confirmed”. With regard to the veracity of compounds compiled from the literature, obviously a different set of rules is required. For those compounds cited and quantified by standard clinical textbooks as well as those identified by two or more independent published studies were considered “confirmed”. Those compounds with only a single literature reference were considered as “probable” unless the presented evidence was overwhelming. These verification labels have been added to all the compound data in the Serum Metabolome Database (see below).

## Supporting Information

File S1 **Supplementary materials and methods information regarding sample preparation, analysis and combinatorial lipid reconstruction.**
(DOC)

Table S1 **Mean and standard deviation (µM) seen over the 12-week sampling period for the 44 metabolites as measured for the 9 heart transplant patients.**
(DOC)

Table S2 **Targeted Lipid Mediator Surrogate Recoveries.**
(DOC)

Table S3 **Concentrations of total fatty acids in healthy serum by Lipomics and compared with the results of Kuriki** ***et al*** **.** [**9**] **.**
(DOC)

Table S4 **Percentage distribution of plasma cholesteryl esters determined by Lipomics, by Kim** ***et al.*** **** [**10**] **, by Vercaemst** ***et al.*** **** [**11**] **and by Ohrvall** ***et al.*** **** [**12**] **.**
(DOC)

Table S5 **Concentrations of phosphatidylcholines in healthy serum (µM) by DFI MS/MS (Biocrates kit).**
(DOC)

Table S6 **Comparison of the concentrations of phosphatidylcholines in healthy serum quantified by DFI MS/MS (Biocrates kit), by Quehenberger et al.** [**13**] **and estimated by CLR (most probable).**
(DOC)

## Footnotes

^1^ http://www.Biocrates.com/images/stories/pdf/Biocrates_Appl.Note_1001-1.pdf
