# Supplementary material for: The Human Serum Metabolome
Source: PLoS One. 2011 Feb 16;6(2):e16957. doi: 10.1371/journal.pone.0016957 (PMC3040193; doi:10.1371/journal.pone.0016957)
Supplement: File S1 — Supplementary materials and methods information regarding sample preparation, analysis and combinatorial lipid reconstruction. (DOC) [file pone.0016957.s001.doc]

# Supporting Information File S1

Nikolaos Psychogios^1^, David D. Hau^1^, Jun Peng^2^, An Chi Guo^1^, Rupasri Mandal^1^, Souhaila Bouatra^1^, Igor Sinelnikov^1^, Ramanarayan Krishnamurthy^1^, Roman Eisner^1^, Bijaya Gautam^1^, Nelson Young^1^, Jianguo Xia^4^, Craig Knox^1^, Edison Dong^1^, Paul Huang^1^, Zsuzsanna Hollander^6^, Theresa L. Pedersen^7^, Steven R. Smith^8^, Fiona Bamforth^3^, Russ Greiner^1^, Bruce McManus^6^, John W. Newman^7^, Theodore Goodfriend^9^, David S. Wishart^1,4,5^

# The Human Serum Metabolome

Departments of Computing Science^1^, Chemistry^2^, Clinical Laboratory Medicine^3^, and Biological Sciences^4^, University of Alberta, Edmonton, AB, Canada T6G 2E8

National Institute for Nanotechnology^5^, 11421 Saskatchewan Drive, Edmonton, AB, Canada T6G 2M9

James Hogg iCAPTURE Centre for Cardiovascular and Pulmonary Research and the NCE CECR Centre of Excellence for Prevention of Organ Failure (PROOF Centre)^6^, 1081 Burrard Street, Vancouver, BC, Canada V6Z 1Y6

USDA, ARS, Western Human Nutrition Research Center^7^, 430 West Health Sciences Drive, Davis, CA, USA, 95616

Pennington Biomedical Research Center^8^, 6400 Perkins Road, Baton Rouge, LA, USA, 70808-4124

Veterans Administration Hospital and University of Wisconsin School of Medicine and Public Health^9^, Madison, WI, USA

Contact Information: David Wishart (ph. 780-492-0383, fax 780-492-1071, email: [david.wishart@ualberta.ca](mailto:david.wishart@ualberta.ca))

## Supplementary Material and Methods:

### NMR Sample Preparation

All serum samples were deproteinized using ultrafiltration. Prior to filtration, two 0.5 mL, 3 KDa cut-off centrifugal filter units (Millipore Microcon YM-3) were rinsed four times each with 0.5 mL of H_2_O, then centrifuged at 11,000 rpm for 1 hour, to remove residual glycerol bound to the filter membranes. Two 150 μL aliquots of each serum sample were then transferred into the two centrifuge filter devices. The samples were then spun at a rate of 11,000 rpm for 140 minutes, to remove macromolecules (primarily proteins and lipoproteins) from the sample. The subsequent filtrates were then checked visually for a red tint, which indicates that the membrane was compromised. For those “membrane compromised” samples, we repeated the filtration process with a different filter and inspected the filtrate again. We then pooled the filtrates that passed the inspections and recorded the volume. If the total volume of the sample was under 300 μL, we added an appropriate amount from a 50 mM NaH_2_PO_4_ buffer (pH 7) to the sample until the total volume was 300 μL. Subsequently, 35 μL of D_2_O and 15 μL of a standard buffer solution (11.667 mM DSS [disodium-2,2-dimethyl-2-silapentane-5-sulphonate], 730 mM imidazole, and 0.47% NaN_3_ in H_2_O) was added to the sample. The serum sample (350 μL) was then transferred to a standard Shigemi microcell NMR tube for subsequent spectral analysis.

### NMR Spectroscopy

All ^1^H-NMR spectra were collected on a 500 MHz Inova (Varian Inc., Palo Alto, CA) spectrometer equipped with either a 5 mm HCN Z-gradient pulsed-field gradient (PFG) room-temperature probe or a Z-gradient PFG Varian cold-probe. ^1^H-NMR spectra were acquired at 25 °C using the first transient of the tnnoesy-presaturation pulse sequence, which was chosen for its high degree of quantitative accuracy [1]. Spectra were collected with 128 transients and 8 steady-state scans using a 4 s acquisition time and a 1 s recycle delay. For certain confirmatory experiments, higher field (800 MHz Varian Inova) instruments, larger numbers of transients (256 or 512) or 2D NMR spectroscopy were used.

### NMR Compound Identification and Quantification

All FIDs were zero-filled to 64k data points and subjected to line broadening of 0.5 Hz. The singlet produced by a known quantity the DSS methyl groups was used as an internal standard for chemical shift referencing (set to 0 ppm) and for quantification. All ^1^H-NMR spectra were processed and analyzed using the Chenomx NMR Suite Professional software package version 6.0 (Chenomx Inc., Edmonton, AB), as previously described . Each spectrum was processed and analyzed by at least two experienced NMR spectroscopists to minimize compound mis-identification and mis-quantification. We also used 2D NMR experiments (TOCSY, COSY) and sample spiking to confirm the identities of a number of ambiguously assigned compounds. Sample spiking involves the addition of 20-200 μM of the suspected compound to the sample of interest and inspection of the subsequent NMR spectra to determine if the expected NMR signals have changed.

### GC-MS Compound Identification and Quantification

Prior to analysis by GC-MS the serum samples were extracted to separate polar metabolites from non-polar (lipophilic) metabolites.

Polar metabolites: Briefly, an aliquot of 100 µL of serum was extracted with 800 µL of cold HPLC grade methanol:double-distilled water (8:1 vol/vol) and vortexed for 1 min. The sample was kept at 4°C for 20 minutes and then centrifuged at 6,000 rpm for 8 minutes. After centrifugation, 200 µL of the supernatant were evaporated to dryness using a Speedvac concentrator (Savant Instruments Inc., SDC-100-H, Farmingdale, NY).

Lipophilic metabolites: Briefly, an aliquot of 200 µL serum was mixed with 1.5 mL CHCl_3_, 0.5 mL MeOH and 300 µl of 0.5 M KH_2_PO_4_, vortexed for 1 min and then centrifuged at 2,500 rpm for 10 minutes. After centrifugation, the supernatant was removed and washed with 300 µL of water. The organic phase was decanted from the aqueous phase and evaporated to dryness using a Speedvac concentrator.

After Speedvac evaporation, a common protocol for carbonyl methoximation and hydroxyl, primary amine and thiol silylation was followed for both the polar and lipohpilic metabolites. Extracted residues were reconstituted in 40 µL of 20 mg/mL methoxyamine hydrochloride (Sigma-Aldrich) in ACS grade pyridine and incubated at room temperature for 16 hours. Then 50 µL of MSTFA (N-Methyl-N-trifluoroacetamide) with 1% TMCS (trimethylchlorosilane) derivatization agent (Pierce) was added and incubated at 37 °C for 1 hr on a hotplate. The samples were vortexed twice during incubation to ensure complete dissolution. Derivatized samples were stored for < 48 hrs at 4 ºC until analysis.

Derivatized extracts were analyzed using an Agilent 5890 Series II GC-MS operating in electron impact (EI) ionization mode. A 2 µL aliquot was injected with a 5:1 split ratio onto a 30 m × 0.25 mm × 0.25 µm DB-5 column (Agilent Technologies). The injector port temperature was held at 250 °C and the helium carrier gas flow rate was set to 1 mL/min at an initial oven temperature of 50 °C. The oven temperature was increased at 10 °C/min to 310 °C for a final run time of 26 min. Full scan spectra (50-500 m/z; 1.7 scans/sec) were acquired after a 6min solvent delay, with an MS ion source temperature of 200°C. Identification and quantification of metabolites was performed as previously described . Briefly, the Automated Mass Spectral Deconvolution and Identification System (AMDIS) spectral deconvolution software (Version 2.62) from NIST (National Institute of Standards and Technology) was used to process the total ion chromatogram and the EI-MS spectra of each GC peak. After deconvolution, the purified mass spectrum of each of the trimethylsilylated metabolites was identified using the NIST MS Search program (version 2.0d) linked to the 2008 NIST mass spectral library (2008). Retention Indices (RIs) were calculated using a C8-C20 alkane mixture solution (Fluka, Sigma-Aldrich) which served as an external alkane standard. Metabolites were identified by matching the EI-MS spectra with those of reference compounds from the NIST library. In AMDIS, each search produces a list of library spectra (“hits”), which is ranked by the similarity to the target spectrum according to a mathematically computed “match factor”. The match factor indicates the likelihood that our spectrum and the reference NIST spectrum arose from the same compound. In the current case, we considered hits with a match factor of >60% and a probability >20%. These cutoff values have been shown to yield consistent results 206 with synthetic mixtures containing known compounds and with samples independently analyzed by NMR. In addition, authenticity checks were performed by using additional published retention index libraries . Peaks having no match to published retention indices and/or no match to the AMDIS GC/MS spectral library were identified, by matching the experimental RI of each metabolite with an in-house RI library (containing 312 TMS-derivatized human metabolites) developed at the University of Alberta . Thirty-five pure standards obtained from the Human Metabolome Library were run through the GC–MS (using the same protocol described above) to confirm their identity. Retention indices and EI spectra were subsequently used for producing external five-point calibration curves (for quantification). Quantification of polar metabolites based on these calibration curves was performed for the 7 serum samples and the pooled normal sample.

### Targeted Profiling of Lipids and Lipid Mediators

*Targeted GC-MS and LC-ESI-MS/MS analysis:* Briefly, a 25 µL plasma aliquot was diluted with 30 µL methanol containing 0.6 nmol cis-10-pentadecenoic acid (C15:1n5) internal standard, vortexed with 125 µL fresh ethereal diazomethane, and held at room temperature for 10 min. Solvents were removed under vacuum and residues were dissolved in 100 µL hexane containing 0.4 nmol methyl tricosanoate (C23:0) internal standard. Extract aliquots (1 µL) were analyzed for fatty acid methyl esters on an Agilent 6890 GC 5973N MSD with a 30 m x 0.25 mm id x 0.25 µm DB-225ms column using splitless injections (0.7 min purge delay) and helium as the carrier gas with the following optimized gradient: Initial 2.0 mL/min held for 5 min, reduced to 1.0 mL/min at 8.3 min, increased to 3.5 mL/min at 18.3 min, increased to 4.0 mL/min at 18.8 min, and held for 21 min. The oven initial temperature of 60 ºC was held 1 min, increased to 235 ºC at 18.5 min, increased to 240 ºC at 19.5 min, and held for 20.5 min (total run time 42 min). The extended hold allowed cholesterol to elute from the columns between injections. The GC inlet and MS interface temperatures were 225 ºC and 240 ºC, respectively. Spectral data was acquired after a 3 min solvent delay in simultaneous selected ion monitoring / full scan (SIM/Scan) mode using the following parameters: Scan 50-400 m/z; SIM, Group 1: (2 to 45 min): 55.1, 67.1, 74.1, 79.1, 368.1 m/z. Quantification was performed using SIM data, with scans used for confirmation of peak purity and results were corrected for surrogate recoveries.

Solid phase extraction (SPE) extraction LC-ESI-MS/MS analysis of non-esterified oxylipins, acylethanolamides, N-acylglycines, and monoacylglycerols: Samples were removed from the -80 ºC and thawed on wet ice. SPE cartridges (60 mg Oasis HLB, Waters Inc.) were placed on a vacuum manifold, washed with 6 mL of methanol and conditioned with 6mL of wash solution (95:5 water/methanol (v/v) with 0.1% acetic acid). Flow was halted on the wetted columns with the liquid meniscus just above the sorbent bed. The following solutions were then added to the cartridge: 10 µL anti-oxidant solution (0.1 mg/mL EDTA, 0.1 mg/mL butylated hydroxyltoluene in 50% MeOH/H_2_O), 10 µL of a methanol solution containing deuterated surrogates representing each quantified chemical class, followed by a 250 µL plasma sample and 2 mL of wash solution to dilute. Samples were then gravity extracted. Additionally, one in-house plasma reference was extracted with each extraction batch, totaling 7 samples. SPE cartridges were washed with 6 mL of wash solution and dried by low vacuum for 30 minutes. Residues were eluted with 0.5 mL methanol followed by 2.0 mL ethyl acetate, into a 5 mL polypropylene cryo tube (Corning, Cat No. 430492) containing 6 µL 30% glycerol in methanol. Extracts were brought just to dryness by vacuum centrifugation and reconstituted in 70 µL of a 100 nM 1-cyclohexyl urea 3-dodecanoic acid (CUDA; Cayman Chemical) and 1-Phenyl 3-hexanoic acid urea (PHAU) (in-house)— in methanol, as internal standards, and vortexed to dissolve. Extracts were cooled over wet ice and filtered by centrifuge with 0.1 µm hydrophilic PVDF filters (Ultrafree-MC, VV 0.1 µm, Millipore). Analytes were separated using an Acquity UPLC (Waters Corp), followed by MS/MS analysis on an ABI 4000 Q-Trap (Applied Biosystems). Oxylipins were detected using negative mode electrospray ionization, while all other residues were detected in positive mode. Compounds were quantified with six point calibration curves using a ratio response to stable isotope surrogates, using the Analyst software package (Applied Biosystems).

### LC-ESI-MS/MS Compound Identification and Quantification

*Sample collection and analysis:*  FAs were extracted from 100μL plasma aliquots using methylene chloride, methanol and water (2:2:1) followed by treatment with 14% boron trifluoride in methanol at 100˚C for ten minutes. The resulting FA methyl esters were analyzed in a GC2010 (Shimadzu, Columbia, MD) using a 100m SP2560 capillary column (Supelco, Bellefonte, PA).

*Oxylipin nomenclature:* The International Union of Pure and Applied Chemistry (IUPAC) has adopted the abbreviations for oxidized fatty acids following the recommendations of Smith *et al*. [6.7]. Briefly, compounds are named using position, number, and standardized abbreviations of functional groups, carbon chain length, and degree of unsaturation. Plural chemical moieties are listed as Di (two), Tr (three), T (four), P (five), He (six). Abbreviations of chemical moieties are: Ep – Epoxide; H – hydroxy; Hp – hydroperoxide; K – keto. Carbon numbers appearing in this report are abbreviated O (octadeca *i.e.* 18), E (eicosa *i.e.* twenty) and Do (docosa *i.e.* 22). Therefore, 14(15)-epoxyeicostri-(5*Z*,8*Z*,11*Z*)-enoic acid is reduced to 14(15)-EpETrE while 9(10)-epoxyoctadec-(12Z)-enoic acid becomes 9(10)-EpOME. Dihydroxy lipids are named similarly, such that 14,15-dihydroxyeicostri-(5*Z*,8*Z*,11*Z*)-enoic acid becomes 14,15-DiHETrE, while 20-hydroxyeicosatetra-(5*Z*,8*Z*,11*Z*,14*Z*)-enoic acid is 20-HETE.

### TLC/GC-FID Lipid Identification and Quantification

This method involves extracting serum lipids in the presence of authentic internal standards via the Folch method [8] using a solution of CHCl_3_/MeOH (2:1 v/v). Individual lipid classes within each extract are separated by preparative thin layer chromatography (TLC) in which the plates are washed/rinsed with solutions containing ethylenediamine tetraacetic acid (EDTA), methanol and chloroform, while various solvent systems including chloroform, methanol, acetic acid, water, petroleum ether and diethyl ether are used as mobile phase. The separated lipids are scraped from the TLC plates and each lipid class is then trans-esterified in 3 N methanolic-HCl in a sealed vial under a nitrogen atmosphere at 100°C for 45 min. The resulting fatty acid methyl esters (FAMEs) are extracted from the mixture with hexane containing 0.05% butylated hydroxytoluene and prepared for GC by sealing the hexane extracts under nitrogen. The resulting FAMEs are separated and quantified by a capillary Hewlett-Packard (Wilmington, DE) 6890 GC instrument equipped with a 30 m DB-225MS or a DB-35MS capillary column (J&W Scientific, Folsom, CA) and a flame ionization detector (FID).

### Combinatorial Lipid Reconstruction

Precise or exact quantification of each of the hundreds of possible fatty acid (FA) combinations within each lipid class (with the exception of cholesterol esters, lysophosphatidylcholines and free FAs, which are lipids that contain only one FA chain) was not possible. Therefore, an alternative indirect scheme that we call Combinatorial Lipid Reconstruction (CLR) was adopted to “reconstruct” glycerolipids and phospholipids with more than one FA chain. As noted earlier, the TrueMass® lipidomics data from Tethys/Lipomics Inc. provided precise concentrations of each lipid class as well as precise concentrations of the constituent (up to 32) FAs from each lipid class. Because lipids are modular in structure (separable head groups and separable tail groups) this information is actually sufficient to reconstruct the lipids and to estimate their concentrations. The concept of CLR is based on using the fractional abundance of each quantified FA moiety as well as on the total abundance of a given lipid class that was calculated across all measured FAs. This combination provided estimations of the “most probable” and the “upper limit” concentrations for each reconstructed lipid. The most probable concentration refers to the concentration a lipid is most likely to have, taking into account all the possible fatty acid combinations within a given lipid class. The upper limit refers to the highest possible concentration a lipid can get without taking into account any other fatty acid combinations within a given lipid class. The average and the standard deviation from three samples were calculated for both the upper limit and the most probable concentrations and listed in the human serum metabolome database (SMDB): [http://www.serummetabolome.ca](http://www.serummetabolome.ca/).

In explaining CLR it is perhaps useful to make an analogy between CLR and polypeptide sequencing via Edman degradation. In chemically based protein sequencing, polypeptides are first cleaved into peptides by proteases, separated or purified and then the short peptides are degraded into individual amino acids (sequenced) via progressive chemical cleavage. The resulting amino acid information is reassembled to create the peptides and the resulting peptides are reassembled to create the complete protein sequence. The reassembled protein sequence is somewhat “theoretical” because the intact protein is actually never sequenced, only its fragments are. The same is true with lipid analysis by CLR. The intact lipid is not fully characterized, only its fragments are. As with protein sequencing (and with CLR) certain assumptions have to be made (cleavage specificity, modular construction of lipids or linear construction of proteins, etc.) and a certain degree of prior knowledge has to be incorporated. More specifically, the underlying assumptions in CLR are that the total number of fatty acid types or varieties in lipids is 32 (the maximum number reported by the Tethys/Lipomics TrueMass® assays). This is reasonable given that these are far-and-away the most abundant (or only) long-chain fatty acids detected in mammals. It also assumes that most lipids have an acyl-linkage as opposed to an alkyl linkage between the lipid head-group and the FAs. This is generally true, but it potentially undercounts the lower abundance alkyl-acyl and alkyl-alkyl classes of lipids that will co-migrate with acyl-acyl lipids. The other assumption in CLR is that fatty-acid acyl transferases exhibit no bias in positional exchange and that lipids exhibit no bias in the preference of fatty acid chains in the sn-1, sn-2 or sn-3 positions. Certainly during lipid biosynthesis there is a bias, but this is moderated by the subsequent random exchange of fatty acids via fatty-acid transferases.

We will use a diacylglycerol (DG) as an example to illustrate the concept behind CLR. A DG is a glyceride consisting of two FA chains covalently bonded to a glycerol molecule through ester linkages. Diacylglycerols can have many different combinations of fatty acids attached at both the sn-1 and sn-2 positions. The TrueMass® platform resulted in the quantification of 25 unique FA chains for DGs. The diglyceride DG(18:2(9Z,12Z)/18:0/0:0) for example, consists of one chain of linoleic acid ((9Z,12Z)-9,12-octadecadienoic acid) at the sn-1 position and one chain of stearic acid (octadecanoic acid; C18:0) at the sn-2 position (see below).


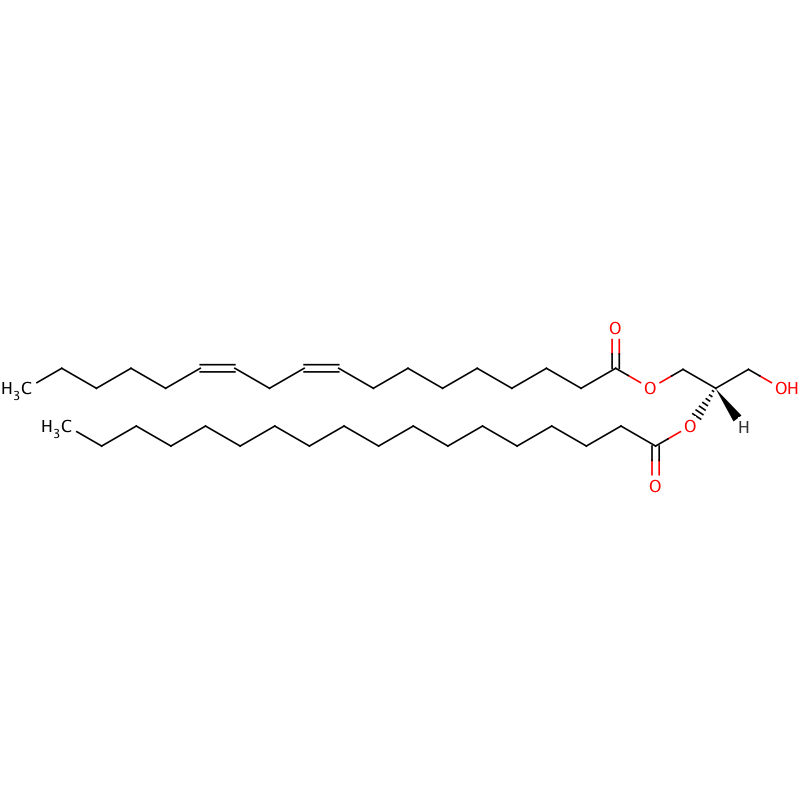


On the other hand, DG(18:0/18:2(9Z,12Z)/0:0) consists of one chain of stearic acid at the sn-1 position and one chain of linoleic acid at the sn-2 position (see below).


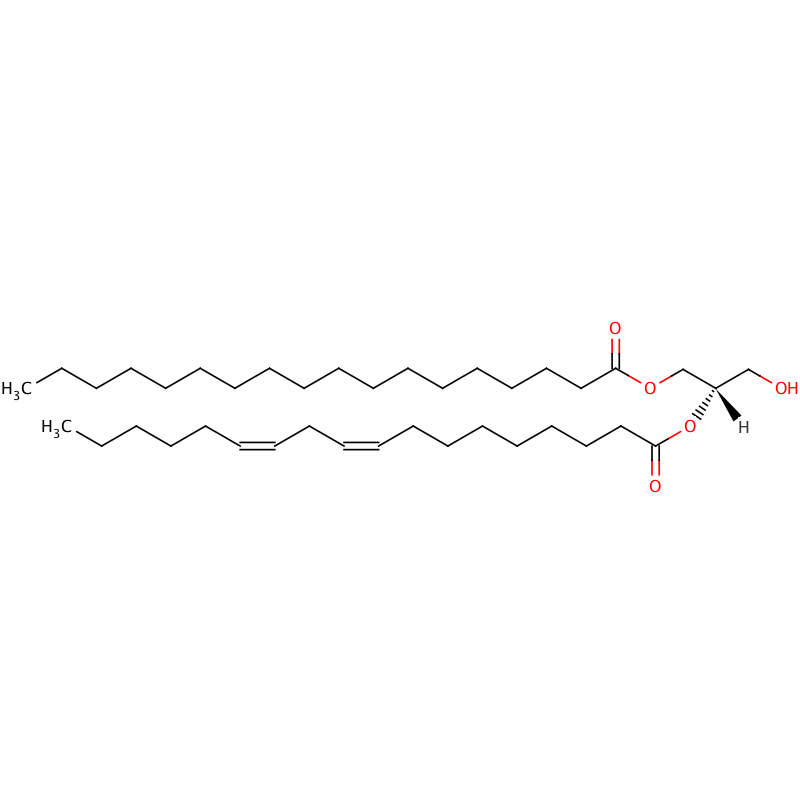


The quantification values of the 25 FAs associated with DGs in the 3 serum samples resulted in an average concentration of 9.13 μM and a fractional abundance of 14.61% for stearic acid and an average concentration of 5.28 μM and a fractional abundance of 8.48% for linoleic acid. Taking into account the concentrations and fractional abundances for the remaining 23 FAs for DGs (which were also measured), the CLR method estimated that the most probable concentration for DG(18:2(9Z,12Z)/18:0/0:0) and DG(18:0/18:2(9Z,12Z)/0:0) is 0.767 +/- 0.190 μM, whereas the upper limit concentration is 10.56 +/- 2.82 μM. The upper limit calculation assumes all the stearic and linoleic acids measured for the DG class have ended up on just these two DGs (a highly unlikely event). Since the TrueMass® platform does not provide any information on the positional distribution of the stearic and linoleic acids in DGs, it is reasonable to estimate that the concentration of DG(18:2(9Z,12Z)/18:0/0:0) and DG(18:0/18:2(9Z,12Z)/0:0) ranges from the most probable to the upper limit concentration, namely from (0.767 - 10.56) μM. If we assume that the most probable concentration is closer to the real one, then the remaining (9.13 – 0.767) = 8.363 μΜ of stearic acid and (5.28 – 0.767) = 4.513 μM of linoleic acid are distributed in the reconstruction of the remaining 23 FAs of DGs.

CLR can also be explained more mathematically. Let [A] be the total molar concentration of all fatty acid chains and let [FA] be the concentration of a given fatty acid chain class. For each available position of lipid head group, if we assume every FA has unbiased chance of being attached to (or cleaved from) a lipid head group, then the probability of a given FA being attached to (or cleaved from) a lipid head group is determined by its relative abundance, that is: p(FA) = [FA]/[A]. Therefore the most probable concentration (MP) of a given lipid can be calculated as follows (using TG with three different side chains - FA1, FA2, and FA3 as an instance): [TG]_MP_ = [A]*p(TG) = [A]* p(FA1)*p(FA2)*p(FA3).

On the other hand, the upper limit or maximal concentration of a given TG structure is determined by the minimum concentration of each of its constituent fatty acids FA1, FA2, FA3: [TG]max = min ([FA1], [FA2], [FA3]).

The same principals described in the above examples were used for the lipid reconstruction and quantification of triacylglycerols, phosphatidylcholines and phosphatidylcholines, which allowed the identification and quantification of 847 “probable” diacylglycerols, 1092 “probable” phosphatidylcholines, 1071 phosphatidylethanolamines, and 289 “probable” triacylglycerols. Lipid identification and quantification based on the combinatorial lipid reconstruction can be performed in the freely available web server: http://www.metaboanalyst.ca/faces/LipidAnalysis.jsp

A more complete or definitive reconstruction of phospholipids would be possible if the exact positional information of FA moieties in the lipid backbone was available. This could be performed with the enzymatic hydrolysis of phospholipids with the use of specific phospholipases (e.g. phospholipase A1 and phospholipase A2) that cleave the FA chains on sn-1 and sn-2 positions. This method is being currently developed in our lab.

### Direct Flow Injection MS/MS Compound Identification and Quantification

This method involves derivatization and extraction of analytes from the biofluid of interest, along with selective mass-spectrometric detection and quantification via multiple reaction monitoring (MRM) using an ion trap mass spectrometer. Isotope-labeled internal standards are integrated into the kit plate filter to facilitate metabolite quantification. A total of twenty normal serum samples (collected via the iCAPTURE study) were analyzed with the AbsoluteIDQ kit using the protocol described in the AbsoluteIDQ user manual. Briefly, serum samples were left to thaw on ice and then vortexed and centrifuged at 13,000 rpm. Ten µL of supernatant from each serum sample was loaded onto the kit’s filter paper substrate and dried in a stream of nitrogen. Extraction of the metabolites was then achieved using methanol containing 5 mM ammonium acetate. The extracts were analyzed using an ABI 4000 Q-Trap (Applied Biosystems/MDS Sciex) mass spectrometer equipped with a solvent delivery system. A standard flow injection protocol consisting of two 20 μL injections (one for the positive and one for the negative ion detection mode) was applied for all measurements. MRM detection was used for quantification. The Biocrates MetIQ software was used to control the entire assay workflow, from sample registration to automated calculation of metabolite concentrations to the export of data into other data analysis programs.

## References

1. Saude EJ, Slupsky CM, Sykes BD (2006) Optimization of NMR analysis of biological fluids for quantitative accuracy. Metabolomics 2: 113-123.

2. Wishart DS, Lewis MJ, Morrissey JA, Flegel MD, Jeroncic K, et al. (2008) The human cerebrospinal fluid metabolome. J Chromatogr B Analyt Technol Biomed Life Sci 871: 164-173.

3. Wishart DS (2008) Quantitative metabolomics using NMR. Trac-Trends in Analytical Chemistry 27: 228-237.

4. Wishart DS, Tzur D, Knox C, Eisner R, Guo AC, et al. (2007) HMDB: the Human Metabolome Database. Nucleic Acids Res 35: D521-526.

5. Wishart DS, Knox C, Guo AC, Eisner R, Young N, et al. (2009) HMDB: a knowledgebase for the human metabolome. Nucleic Acids Res 37: D603-610.

6. Smith DL, Willis AL (1987) A suggested shorthand nomenclature for the eicosanoids. Lipids 22: 983-986.

7. Smith WL, Borgeat P, Hamberg M, Roberts LJ, 2nd, Willis A, et al. (1990) Nomenclature. Methods Enzymol 187: 1-9.

8. Folch J, Lees M, Sloane Stanley GH (1957) A simple method for the isolation and purification of total lipides from animal tissues. J Biol Chem 226: 497-509.

9. Kuriki K, Nagaya T, Tokudome Y, Imaeda N, Fujiwara N, et al. (2003) Plasma concentrations of (n-3) highly unsaturated fatty acids are good biomarkers of relative dietary fatty acid intakes: a cross-sectional study. J Nutr 133: 3643-3650.

10. Kim DH, Lee KJ, Heo GS (1994) Analysis of cholesterol and cholesteryl esters in human serum using capillary supercritical fluid chromatography. J Chromatogr B Biomed Appl 655: 1-8.

11. Vercaemst R, Union A, Rosseneu M, De Craene I, De Backer G, et al. (1989) Quantitation of plasma free cholesterol and cholesteryl esters by high performance liquid chromatography. Study of a normal population. Atherosclerosis 78: 245-250.

12. Ohrvall M, Berglund L, Salminen I, Lithell H, Aro A, et al. (1996) The serum cholesterol ester fatty acid composition but not the serum concentration of alpha tocopherol predicts the development of myocardial infarction in 50-year-old men: 19 years follow-up. Atherosclerosis 127: 65-71.

13. Quehenberger O, Armando AM, Brown AH, Milne SB, Myers DS, et al. (2010) Lipidomics reveals a remarkable diversity of lipids in human plasma. J Lipid Res 51: 3299-3305.
