# Supplementary material for: The Human Serum Metabolome
Source: PLoS One. 2011 Feb 16;6(2):e16957. doi: 10.1371/journal.pone.0016957 (PMC3040193; doi:10.1371/journal.pone.0016957)
Supplement: Table S1 — Mean and standard deviation (µM) seen over the 12-week sampling period for the 44 metabolites as measured for the 9 heart transplant patients. (DOC) [file pone.0016957.s002.doc]

**Table S1.** Mean and standard deviation (μM) seen over the 12-week sampling period for the 44 metabolites as measured for the 9 heart transplant patients

|  | Cross-sectional | | | Longitudinal | | |
| --- | --- | --- | --- | --- | --- | --- |
|  | Average | St. Dev. | Variation | Average | St. Dev. | Variation |
| **Isobutyric acid** | 8.4 | 1.9 | 23.1 | 7.6 | 1.4 | 18.5 |
| Choline | 9.7 | 4.5 | 46.6 | 9.9 | 2.8 | 28.4 |
| **Alpha-ketoisovaleric acid** | 10.7 | 5.5 | 51.6 | 11.5 | 3.6 | 31.5 |
| Methylmalonic acid | 11.2 | - | - | 11.2 | - | - |
| Acetone | 13.2 | 5.5 | 47.1 | 13.1 | 5.5 | 47.1 |
| **Isopropyl alcohol** | 16.5 | 22.5 | 136.4 | 14.6 | 9.3 | 63.5 |
| L-Methionine | 17.3 | 9.5 | 55.1 | 17.1 | 5.2 | 30.2 |
| Formic acid | 19.6 | 6.8 | 34.5 | 18.9 | 3.5 | 18.7 |
| 2-Hydroxybutyric acid | 24.3 | 14.5 | 59.9 | 24.5 | 7.3 | 29.9 |
| Acetoacetic acid | 27.3 | 14.4 | 52.6 | 28.3 | 14.5 | 51.0 |
| Acetaminophen | 33.5 | 22.3 | 66.7 | 30.2 | 12.2 | 40.3 |
| Creatine | 33.8 | 37.7 | 111.6 | 33.4 | 25.4 | 75.8 |
| 3-Hydroxybutyric acid | 35.1 | 33.9 | 96.6 | 35.5 | 14.2 | 40.1 |
| Propylene glycol | 36.3 | 19.9 | 54.9 | 32.1 | 15.2 | 47.2 |
| Ethanol | 40.2 | 12.1 | 30.0 | 39.6 | 13.0 | 32.8 |
| L-Carnitine | 41.7 | 23.9 | 57.2 | 41.9 | 14.1 | 33.6 |
| Betaine | 42.1 | 19.3 | 45.8 | 42.1 | 9.9 | 23.4 |
| Acetic acid | 42.2 | 17.3 | 41.0 | 42.3 | 10.0 | 23.6 |
| L-Isoleucine | 44.6 | 21.5 | 48.2 | 44.7 | 11.7 | 26.2 |
| L-Phenylalanine | 44.8 | 21.0 | 46.9 | 45.0 | 12.7 | 28.1 |
| L-Histidine | 46.1 | 17.5 | 37.9 | 46.0 | 9.8 | 21.2 |
| Pyruvic aicd | 50.2 | 40.0 | 79.7 | 47.6 | 21.4 | 44.9 |
| Xanthine | 51.2 | - | - | 51.2 | - | - |
| Hypoxanthine | 52.3 | - | - | 52.3 | - | - |
| L-Asparagine | 54.1 | 21.7 | 40.1 | 52.3 | 18.0 | 34.3 |
| L-Tyrosine | 57.2 | 24.4 | 42.6 | 57.0 | 10.5 | 18.5 |
| L-Ornithine | 65.4 | 30.4 | 46.5 | 65.0 | 18.8 | 28.9 |
| L-Glutamic acid | 72.0 | 36.9 | 51.3 | 64.3 | 29.3 | 45.6 |
| L-Leucine | 74.8 | 34.3 | 45.8 | 74.8 | 20.4 | 27.3 |
| Citric acid | 80.0 | 44.9 | 56.1 | 80.2 | 17.4 | 21.6 |
| Methanol | 81.5 | 55.2 | 67.8 | 80.9 | 16.7 | 20.7 |
| L-Threonine | 83.4 | 47.8 | 57.4 | 82.6 | 19.2 | 23.2 |
| Creatinine | 86.9 | 44.5 | 51.3 | 87.6 | 28.0 | 32.0 |
| Malonic acid | 105.7 | 95.8 | 90.7 | 84.7 | 57.2 | 67.5 |
| L-Lysine | 128.2 | 55.3 | 43.2 | 128.0 | 26.9 | 21.0 |
| Glycerol | 133.9 | 87.8 | 65.5 | 134.6 | 35.0 | 26.0 |
| L-Valine | 144.2 | 61.4 | 42.6 | 144.0 | 38.4 | 26.7 |
| L-Proline | 159.9 | 86.3 | 54.0 | 159.4 | 52.5 | 32.9 |
| Glycine | 234.9 | 181.1 | 77.1 | 234.0 | 87.1 | 37.2 |
| L-Alanine | 340.0 | 126.2 | 37.1 | 341.0 | 68.3 | 20.0 |
| L-Glutamine | 376.8 | 114.3 | 30.3 | 376.8 | 55.7 | 14.8 |
| L-Lactic acid | 1401.2 | 692.1 | 49.4 | 1409.6 | 338.8 | 24.0 |
| Urea | 3309.9 | 1844.0 | 55.7 | 3302.0 | 895.4 | 27.1 |
| D-Glucose | 3743.0 | 1272.9 | 34.0 | 3752.9 | 658.2 | 17.5 |
