# Supplementary material for: The Human Serum Metabolome
Source: PLoS One. 2011 Feb 16;6(2):e16957. doi: 10.1371/journal.pone.0016957 (PMC3040193; doi:10.1371/journal.pone.0016957)
Supplement: Table S2 — Targeted Lipid Mediator Surrogate Recoveries. (DOC) [file pone.0016957.s003.doc]

**Table S2**: Targeted Lipid Mediator Surrogate Recoveries

| **Surrogate**  **Recoveries** | **Pennington (Plasma + LRM)**  **(n=77)** | **Human Metabolome**  **Plasma (n=4)** |
| --- | --- | --- |
| d4 6-keto PGF1a | 65 ± 5% | 94 ± 5% |
| d4-TXB2 | 68 ± 7% | 88 ± 19% |
| d4-PGE2 | 66 ± 7% | 106 ± 22% |
| d4-PGD2 | 61 ± 4% | 110 ± 24% |
| d4-LTB4 | 67 ± 5% | 81 ± 8% |
| d11-14,15-DiHETrE | 86 ± 4% | 48 ± 6% |
| d6-20-HETE | 64 ± 4% | 31 ± 6% |
| d4-9(S)-HODE | 64 ± 3% | 52 ± 10% |
| d8-12(S)-HETE | 46 ± 2% | 62 ± 9% |
| d8-5(S)-HETE | 64 ± 3% | 48 ± 5% |
| d8-11(12)-EpETrE | 49 ± 2% | 53 ± 4% |
| d4-PGF2a EA | 79 ± 14% | NA |
| d8-AEA | 36 ± 14% | NA |
| d5-2-AG | 71 ± 23% | NA |
| d8-NA-Gly | 56 ± 31% | NA |

LRM = laboratry reference material
