# Supplementary material for: The Human Serum Metabolome
Source: PLoS One. 2011 Feb 16;6(2):e16957. doi: 10.1371/journal.pone.0016957 (PMC3040193; doi:10.1371/journal.pone.0016957)
Supplement: Table S3 — Concentrations of total fatty acids in healthy serum by Lipomics and compared with the results of Kuriki et al . [9] . (DOC) [file pone.0016957.s004.doc]

**Table S3.** Concentrations of total fatty acids in healthy serum by Lipomics and compared with the results of Kuriki *et al*.

|  | **Mean ± SD (mmol/L)** | | |
| --- | --- | --- | --- |
|  | Lipomics | Kuriki et al | |
|  | **Both genders** | **Males** | **Females** |
| **SFA: (14:0+16:0+18:0)** | **3.20 ± 0.31** | **3.21 ± 0.53** | **3.42 ± 0.82** |
| C14:0 | 0.19 **±** 0.06 | 0.09 **±** 0.02 | 0.09 **±** 0.04 |
| C16:0 | 2.19 **±** 0.29 | 2.36 **±** 0.43 | 2.50 **±** 0.63 |
| C18:0 | 0.82 **±** 0.08 | 0.77 **±** 0.11 | 0.83 **±** 0.16 |
| **MUFA: (16:1+18:1)** | **2.22 ± 0.20** | **2.41 ± 0.66** | **2.25 ± 0.63** |
| C16:1* | 0.20 **±** 0.05 | 0.25 **±** 0.07 | 0.24 **±** 0.10 |
| C18:1** | 2.01 **±** 0.20 | 2.17 **±** 0.60 | 2.01 **±** 0.53 |
| **(n-6) PUFA: [18:2n6+20:4n6]** | **3.39 ± 0.32** | **4.00 ± 0.66** | **4.15 ± 0.71** |
| C18:2n6 (LA) | 2.75 **±** 0.32 | 3.25 **±** 0.60 | 3.36 **±** 0.63 |
| C20:4n6 (AA) | 0.64 **±** 0.03 | 0.59 **±** 0.12 | 0.66 **±** 0.13 |
| **(n-3)PUFA: 18:3n3 (ALA)** | 0.06 **±** 0.01 | 0.10 **±** 0.04 | 0.08 **±** 0.03 |
| **(n-3)HUFA: [20:5n3+22:5n3+22:6n3]** | **0.40 ± 0.0** | **0.68 ± 0.23** | **0.81 ± 0.24** |
| C20:5n3 (EPA) | 0.12 **±** 0.02 | 0.20 **±** 0.11 | 0.26 **±** 0.11 |
| C22:5n3 | 0.06 **±** 0.01 | 0.06 **±** 0.02 | 0.06 **±** 0.02 |
| C22:6n3 (DHA) | 0.23 **±** 0.04 | 0.42 **±** 0.11 | 0.49 **±** 0.13 |
| **Total FA** | **9.27 ± 0.49** | **10.40 ± 1.83** | **10.71 ± 2.21** |

*; 16:1 corresponds to 16:1n7 in the Lipomics panel

**; 18:1 corresponds to the sum of 18:1n7 and 18:1n9 in the Lipomics panel

Abbreviations: AA, arachidonic acid; ALA, alpha linolenic acid; DHA, docosahexaenoic acid; EPA, eicosapentaenoic acid; FA, fatty acid; HUFA, highly unsaturated fatty acid; LA, linoleic acid; MUFA, monounsaturated fatty acid; PUFA, polyunsaturated fatty acid SFA, saturated fatty acid
