# Supplementary material for: The Human Serum Metabolome
Source: PLoS One. 2011 Feb 16;6(2):e16957. doi: 10.1371/journal.pone.0016957 (PMC3040193; doi:10.1371/journal.pone.0016957)
Supplement: Table S4 — Percentage distribution of plasma cholesteryl esters determined by Lipomics, by Kim et al. [10] , by Vercaemst et al. [11] and by Ohrvall et al. [12] . (DOC) [file pone.0016957.s005.doc]

**Table S4.** Percentage distribution of plasma cholesteryl esters determined by Lipomics, by Kim *et al.* [10], by Vercaemst *et al.*[11] and by Ohrvall *et al.* [12]

|  | **Mean ± SD (% composition)** | | | | |
| --- | --- | --- | --- | --- | --- |
|  | Lipomics | Kim *et al.* [10] | Vercaemst *et al*  [11] | | Ohrvall *et al. [*[12] |
|  | Both genders | **-** | **Males** | Females | **Males** |
| C14:0 | 3.07 ± 1.94 | 11 | - | - | 1.13 ± 0.25 |
| C16:0 | 12.44 ± 0.48 | 8 | 13.3 ± 2.1 | 12.5 ± 2.1 | 11.65 ± 0.97 |
| C16:1n7 | 3.6 ± 1.0 | 5 | - | - | 3.83 ± 1.36 |
| C18:0 | 1.16 ± 0.08 | 1 | - | - | 1.15 ± 0.28 |
| C18:1n9 | 21.52 ± 164 | - | 21.9 ± 4.5 | 22.0 ± 3.6 | 19.38 ± 2.77 |
| C18:2n6 | 46.19 ± 0.57 | 45 | 58.8 ± 5.9 | 59.5 ± 5.0 | 54.1 ± 5.24 |
| C18:3n6 | 0.73 ± 0.07 |  |  |  | 0.70 ± 0.29 |
| C18:3n3 | 0.73 ± 0.11 |  |  |  | 0.66 ± 0.16 |
| C20:3n6 | 0.58 ± 0.16 |  |  |  | 0.57 ± 0.13 |
| C20:4n6 | 6.05 ± 0.85 | 11 | 5.9 ± 1.2 | 6.0 ± 1.2 | 4.77 ± 0.97 |
| C20:5n3 | 1.18 ± 0.19 |  |  |  | 1.35 ± 0.62 |
| C22:6n3 | 0.66 ± 0.13 |  |  |  | 0.70 ± 0.21 |
| Total | 2120 ± 290 (µg/mL) | 1532 ± 14 (µg/mL) |  |  |  |
