# Supplementary material for: The Human Serum Metabolome
Source: PLoS One. 2011 Feb 16;6(2):e16957. doi: 10.1371/journal.pone.0016957 (PMC3040193; doi:10.1371/journal.pone.0016957)
Supplement: Table S5 — Concentrations of phosphatidylcholines in healthy serum (µM) by DFI MS/MS (Biocrates kit). (DOC) [file pone.0016957.s006.doc]

**Table S5.** Concentrations of phosphatidylcholines in healthy serum (μΜ) by DFI MS/MS (Biocrates kit)

| **Phosphatidylcholines** | | | | | | | | |
| --- | --- | --- | --- | --- | --- | --- | --- | --- |
|  | **Mean** | **SD** |  | **Mean** | **SD** |  | **Mean** | **SD** |
| **PC aa C28:1** | 2.37 | 0.67 | **PC aa C40:3** | 0.774 | 0.154 | **PC ae C38:0** | 2.08 | 0.73 |
| **PC aa C30:0** | 4.00 | 1.07 | **PC aa C40:4** | 3.67 | 1.10 | **PC ae C38:1** | 4.96 | 1.29 |
| **PC aa C30:2** | 3.76 | 0.88 | **PC aa C40:5** | 11.29 | 3.58 | **PC ae C38:2** | 5.14 | 1.07 |
| **PC aa C32:0** | 10.83 | 2.50 | **PC aa C40:6** | 23.6 | 7.3 | **PC ae C38:3** | 4.37 | 1.06 |
| **PC aa C32:2** | 5.95 | 1.75 | **PC aa C42:0** | 0.489 | 0.172 | **PC ae C38:4** | 10.56 | 2.35 |
| **PC aa C32:3** | 0.474 | 0.111 | **PC aa C42:1** | 0.241 | 0.090 | **PC ae C38:5** | 14.3 | 2.8 |
| **PC aa C34:1** | 195 | 49 | **PC aa C42:2** | 0.172 | 0.056 | **PC ae C38:6** | 6.18 | 1.21 |
| **PC aa C34:2** | 307 | 58 | **PC aa C42:4** | 0.175 | 0.052 | **PC ae C40:1** | 1.70 | 0.40 |
| **PC aa C34:3** | 14.46 | 5.04 | **PC aa C42:5** | 0.322 | 0.081 | **PC ae C40:2** | 1.63 | 0.41 |
| **PC aa C34:4** | 1.37 | 0.52 | **PC aa C42:6** | 0.513 | 0.090 | **PC ae C40:3** | 1.31 | 0.54 |
| **PC aa C36:0** | 5.89 | 1.16 | **PC ae C30:0** | 0.294 | 0.069 | **PC ae C40:4** | 2.36 | 0.73 |
| **PC aa C36:1** | 63.1 | 12.6 | **PC ae C30:1** | 1.49 | 0.43 | **PC ae C40:5** | 3.88 | 1.19 |
| **PC aa C36:2** | 218 | 39 | **PC ae C30:2** | 0.064 | 0.019 | **PC ae C40:6** | 4.26 | 0.98 |
| **PC aa C36:3** | 132 | 27 | **PC ae C32:1** | 2.73 | 0.54 | **PC ae C42:0** | 0.437 | 0.040 |
| **PC aa C36:4** | 145.63 | 40.50 | **PC ae C32:2** | 0.483 | 0.093 | **PC ae C42:1** | 0.284 | 0.075 |
| **PC aa C36:5** | 24.04 | 12.27 | **PC ae C34:0** | 1.70 | 0.40 | **PC ae C42:2** | 0.500 | 0.107 |
| **PC aa C36:6** | 1.043 | 0.348 | **PC ae C34:1** | 7.65 | 1.38 | **PC ae C42:3** | 0.663 | 0.168 |
| **PC aa C38:0** | 3.00 | 0.67 | **PC ae C34:2** | 8.77 | 1.97 | **PC ae C42:4** | 0.943 | 0.300 |
| **PC aa C38:1** | 8.53 | 2.33 | **PC ae C34:3** | 6.41 | 1.96 | **PC ae C42:5** | 1.80 | 0.52 |
| **PC aa C38:3** | 47.3 | 12.9 | **PC ae C36:0** | 1.21 | 0.23 | **PC ae C44:3** | 0.130 | 0.037 |
| **PC aa C38:4** | 84.5 | 25.2 | **PC ae C36:1** | 7.67 | 1.76 | **PC ae C44:4** | 0.506 | 0.151 |
| **PC aa C38:5** | 51.6 | 15.4 | **PC ae C36:2** | 11.9 | 2.4 | **PC ae C44:5** | 1.61 | 0.52 |
| **PC aa C38:6** | 62.8 | 17.5 | **PC ae C36:3** | 8.11 | 1.69 | **PC ae C44:6** | 1.10 | 0.42 |
| **PC aa C40:1** | 0.511 | 0.086 | **PC ae C36:4** | 13.9 | 3.1 |  |  |  |
| **PC aa C40:2** | 0.462 | 0.120 | **PC ae C36:5** | 9.3 | 2.6 |  |  |  |

**aa: diacyl; ae: acyl-alkyl**
