# Supplementary material for: The Human Serum Metabolome
Source: PLoS One. 2011 Feb 16;6(2):e16957. doi: 10.1371/journal.pone.0016957 (PMC3040193; doi:10.1371/journal.pone.0016957)
Supplement: Table S6 — Comparison of the concentrations of phosphatidylcholines in healthy serum quantified by DFI MS/MS (Biocrates kit), by Quehenberger et al. [13] and estimated by CLR (most probable). (DOC) [file pone.0016957.s007.doc]

**Table S6. Comparison of the concentrations of phosphatidylcholines in healthy serum quantified by DFI MS/MS (Biocrates kit), by Quehenberger et al. [13] and estimated by CLR (most probable).**

|  | **DFI MS/MS** | | **CLR** | | **Quehenberger *et al.* [13]** | |
| --- | --- | --- | --- | --- | --- | --- |
| **PC** | **Mean** | **SD** | **Mean** | **SD** | Mean | **SEM** |
| **C28:1** | 2.37 | 0.67 | 0.006 | 0.004 | N/A | N/A |
| **C30:0** | 4.00 | 1.07 | 9.97 | 2.54 | N/A | N/A |
| **C30:2** | 3.76 | 0.88 | 0.010 | 0.007 | N/A | N/A |
| **C32:0** | 10.83 | 2.50 | 356.80 | 91.57 | 11.4 | 0.6 |
| **C32:2** | 5.95 | 1.75 | 8.28 | 2.38 | 9.80 | 0.45 |
| **C32:3** | 0.474 | 0.111 | 0.480 | 0.275 | N/A | N/A |
| **C34:1** | 195 | 49 | 334.05 | 86.12 | 89.3 | 8.1 |
| **C34:2** | 307 | 58 | 572.71 | 161.22 | 188 | 14 |
| **C34:3** | 14.46 | 5.04 | 21.88 | 10.33 | 13.8 | 1.0 |
| **C34:4** | 1.37 | 0.52 | 3.16 | 0.38 | N/A | N/A |
| **C36:0** | 5.89 | 1.16 | 35.86 | 4.24 | 7.95 | 1.51 |
| **C36:1** | 63.1 | 12.6 | 148.79 | 27.29 | 99.8 | 13.0 |
| **C36:2** | 218 | 39 | 331.31 | 72.62 | 254 | 18 |
| **C36:3** | 132 | 27 | 327.07 | 81.02 | 165 | 13 |
| **C36:4** | 145.63 | 40.50 | 316.53 | 52.82 | 172 | 11 |
| **C36:5** | 24.04 | 12.27 | 47.39 | 14.47 | 12.8 | 1.2 |
| **C36:6** | 1.043 | 0.348 | 2.234 | 0.802 | N/A | N/A |
| **C38:0** | 3.00 | 0.67 | 0.75 | 0.22 | N/A | N/A |
| **C38:1** | 8.53 | 2.33 | 5.95 | 5.37 | N/A | N/A |
| **C38:3** | 47.3 | 12.9 | 34.78 | 4.47 | N/A | N/A |
| **C38:4** | 84.5 | 25.2 | 131.24 | 10.88 | 254 | 21 |
| **C38:5** | 51.6 | 15.4 | 184.89 | 19.58 | 86.3 | 9.0 |
| **C38:6** | 62.8 | 17.5 | 272.67 | 48.07 | 62.9 | 4.9 |
| **C40:1** | 0.511 | 0.086 | 1.80 | 2.34 | N/A | N/A |
| **C40:2** | 0.462 | 0.120 | 1.658 | 2.089 | 133 | 16 |
| **C40:3** | 0.774 | 0.154 | 2.552 | 3.407 | N/A | N/A |
| **C40:4** | 3.67 | 1.10 | 4.57 | 1.86 | 36.7 | 5.5 |
| **C40:5** | 11.29 | 3.58 | 18.56 | 2.97 | 66.6 | 10.1 |
| **C40:6** | 23.6 | 7.3 | 65.13 | 13.44 | 79.4 | 10.3 |
| **C42:0** | 0.489 | 0.172 | 0.181 | 0.074 | N/A | N/A |
| **C42:1** | 0.241 | 0.090 | 0.283 | 0.130 | N/A | N/A |
| **C42:2** | 0.172 | 0.056 | 0.453 | 0.228 | N/A | N/A |
| **C42:4** | 0.175 | 0.052 | 0.398 | 0.480 | N/A | N/A |
| **C42:5** | 0.322 | 0.081 | 1.203 | 1.678 | N/A | N/A |
| **C42:6** | 0.513 | 0.090 | 0.653 | 0.430 | N/A | N/A |
